# Engineering a Surfactant Trap via Postassembly Modification of an Imine Cage

**DOI:** 10.1021/acs.chemmater.4c01808

**Published:** 2024-09-04

**Authors:** María Pérez-Ferreiro, Quinn M. Gallagher, Andrea B. León, Michael A. Webb, Alejandro Criado, Jesús Mosquera

**Affiliations:** † Universidade da Coruña, CICA—Centro Interdisciplinar de Química e Bioloxía, Rúa as Carballeiras, 15071 A Coruña, Spain; ‡ Department of Chemical and Biological Engineering, Princeton University, Princeton, New Jersey 08544, United States

## Abstract

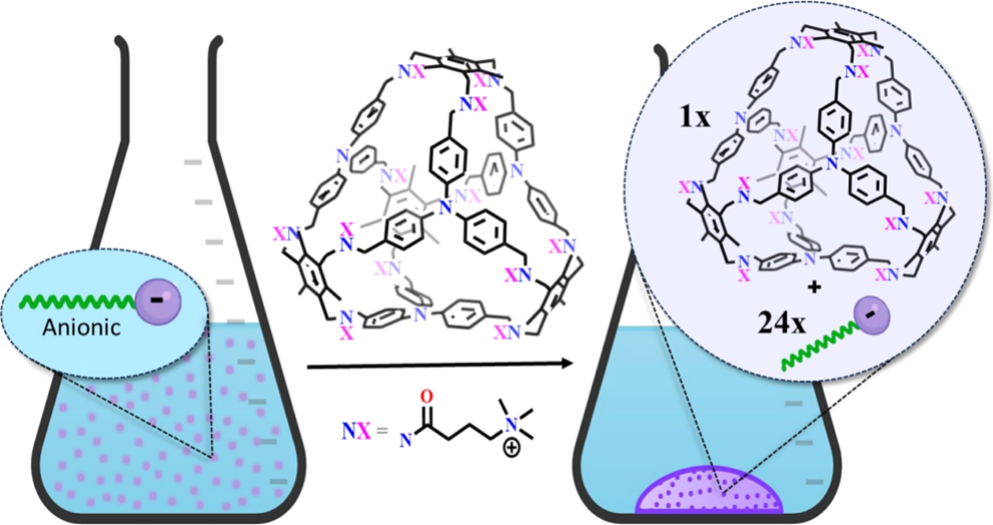

Imine self-assembly stands as a potent strategy for the preparation of molecular organic cages. However, challenges persist, such as water insolubility and limited recognition properties due to constraints in the application of specific components during the self-assembly process. In this study, we addressed these limitations by initially employing a locking strategy, followed by a postassembly modification. This sequential approach enables precise control over both the solubility and host–guest properties of an imine-based cage. The resulting structure demonstrates water solubility and exhibits an exceptional capacity to selectively interact with anionic surfactants, inducing their precipitation. Remarkably, each cage precipitates 24 equiv of anionic surfactants even at concentrations much lower than the surfactant’s critical micelle concentration (CMC), ensuring their complete removal. Molecular simulations elucidate how anionic surfactants specifically interact with the cage to facilitate aggregation below the surfactant CMC and induce precipitation as a micellar cross-linker. This innovative class of cages paves the way for the advancement of materials tailored for environmental remediation.

## 1 Introduction

The field of molecular cages is gaining substantial attention,^1−6^ fueled by the tremendous potential they offer for biological applications,^7−10^ catalysis,^11−13^ and chemical separations.^14,15^ Molecular self-assembly based on imine condensation has emerged as one of the most effective approaches for synthesizing organic cages.^16−18^ However, three major challenges have hindered their use: low solubility in aqueous media,^19,20^ the imine bond instability in the presence of nucleophiles and water,^21,22^ and the difficulties associated with using polar groups in the self-assembly process. Consequently, these cages have mainly been employed in the design of porous solid materials for gas separation and adsorption.^23,24^

The lability of imine bonds can be addressed by employing postassembly covalent locking, converting imines to secondary amino groups.^25−27^ However, addressing the challenge of poor water solubility proves more complex.^28,29^ This issue arises from the need to incorporate rigid aromatic components into the self-assembly process to generate cavities, contributing to the hydrophobic nature of the resulting cages.^30,31^ Additionally, the sensitivity of the imine self-assembly process to polar functionalities restricts the inclusion of polar groups in the cage, limiting both the solubility and molecular recognition.

Surfactants, commonly referred to as “amphiphiles”, are one of the most applied supramolecular units in aqueous media for biological and industrial applications.^32,33^ These molecules consist of a hydrophobic tail and a hydrophilic head. The hydrophobic part is an aliphatic tail, whereas the polar head can vary according to its charge: nonionic, cationic, anionic, or zwitterionic. The amphiphilic nature of these molecules enables them to self-assemble into supramolecular aggregates, called micelles. As a result of their self-assembly properties in aqueous solutions, surfactants have an extensive range of industrial applications. In fact, they are anticipated to exceed a global market value of $52 billion by 2025.^34^

Surfactants present a significant environmental risk because of their impact on water, animal, and vegetal life. Additionally, some surfactants also have severe health implications on humans through ingestion or drinking of contaminated food items.^34^ A promising avenue to remove surfactants is the use of synthetic host molecules that can selectively bind to surfactants. However, research in this area is limited, with only a few studies reporting that traditional hosts like cyclodextrins are capable of encapsulating a single molecule of surfactant without inducing precipitation.^35,36^ Furthermore, the research team led by Sessler and Chi has recently utilized molecular cages to fabricate solid materials designed for the removal of fluorinated surfactants.^37,38^

**Figure 1:**
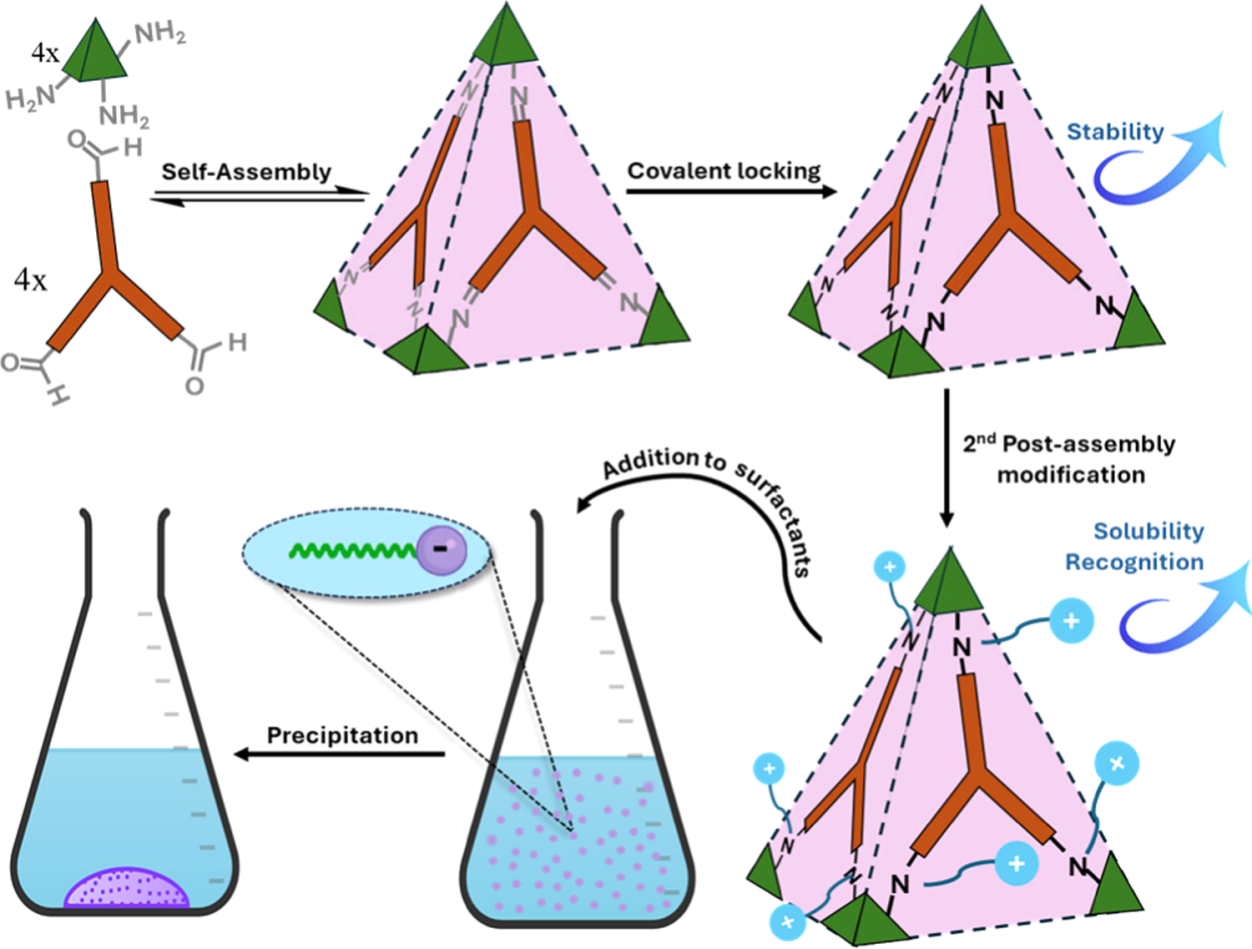
Schematic illustration of the synthetic approach to develop a molecular cage capable of precipitating anionic surfactants.

Herein, we investigate the potential of a secondary postassembly modification^39,40^ of imine-locked structures to enhance water solubility and induce novel recognition properties (Figure 1). Our research suggests that amide bond formation represents a suitable approach for attaching functionalities to the amino groups formed through postassembly covalent locking. By implementing this approach, we achieved the successful synthesis of a functionalized cage that exhibits solubility in both water and phosphate buffer. Furthermore, this cage displayed a distinctive selectivity toward anionic surfactants, exhibiting a unique behavior by serving as a template agent for the formation of insoluble anionic micelles, resulting in the complete elimination of surfactants from the aqueous solution.

## 2 Results and Discussion

### 2.1 Synthesis and Characterization of **A**_**4**_**B**_**4**_ Cage

**Figure 2:**
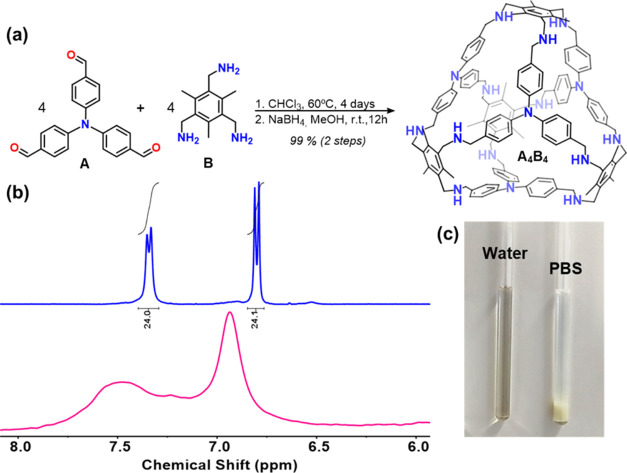
Synthesis and characterization of molecular cage **A**_**4**_**B**_**4**_. (a) Preparation of the molecular cage. (b) ^1^H NMR of **A**_**4**_**B**_**4**_ in DMSO (blue) and D_2_O (pink), showing one set of signals from the aromatic panel, where the broadening of the signals in water due to slow rotation can be observed. (c) Comparison of the solubility of **A**_**4**_**B**_**4**_ in both acidic water and PBS buffer.

Molecular cage **A**_**4**_**B**_**4**_ was prepared by using a one-pot Schiff base condensation involving 1,3,5-tris(aminomethyl)-2,4,6-trimethylbenzene (**A**) and tris(4-formylphenyl)amine (**B**). This process resulted in the formation of a tetrahedral imine-based cage with a cavity diameter of 10 Å, as previously reported by Cooper’s group.^41^ We found that this cage can undergo direct reduction in a one-pot reaction with the addition of sodium borohydride in methanol (Figure 2a). Nuclear magnetic resonance (NMR) and liquid chromatography-mass spectrometry (LC-MS) analyses unequivocally confirmed the successful formation of the covalent-locked cage **A**_**4**_**B**_**4**_ in quantitative yield (Figures S3–S8).

The solubility of **A**_**4**_**B**_**4**_ was assessed in both organic and aqueous solvents. When deprotonated, the cage is insoluble in common solvents and only partially soluble in dimethylformamide (DMF). However, upon protonation with either HCl or trifluoroacetic acid (TFA), **A**_**4**_**B**_**4**_ displays substantially enhanced solubility in water, dimethyl sulfoxide (DMSO), and DMF. Consequently, ^1^H NMR spectroscopy could be utilized for further characterization of this compound. In deuterated DMSO, **A**_**4**_**B**_**4**_ exhibits only one set of sharp ligand resonances (Figure 2b). This can be attributed to the rapid rotation of the aromatic rings within the cage, leading to a highly symmetric structure. Conversely, the ^1^H NMR spectrum of the cage in acidic D_2_O displays significantly broadened signals, suggesting a collapsed structure induced by the hydrophobic effect (Figure 2b).

To determine the potential utility of **A**_**4**_**B**_**4**_ in biological applications, its solubility was examined in phosphate-buffered saline (PBS) at neutral pH. Unfortunately, the cage was found to be insoluble in this medium (Figure 2c). This finding suggests that while the presence of secondary amino groups in organic cages enhances solubility in acidic conditions through protonation, this effect alone is insufficient to confer solubility at neutral pH. This may explain why the study of recognition properties of reduced organic cages has been limited to organic solvents.^42^

**Figure 3:**
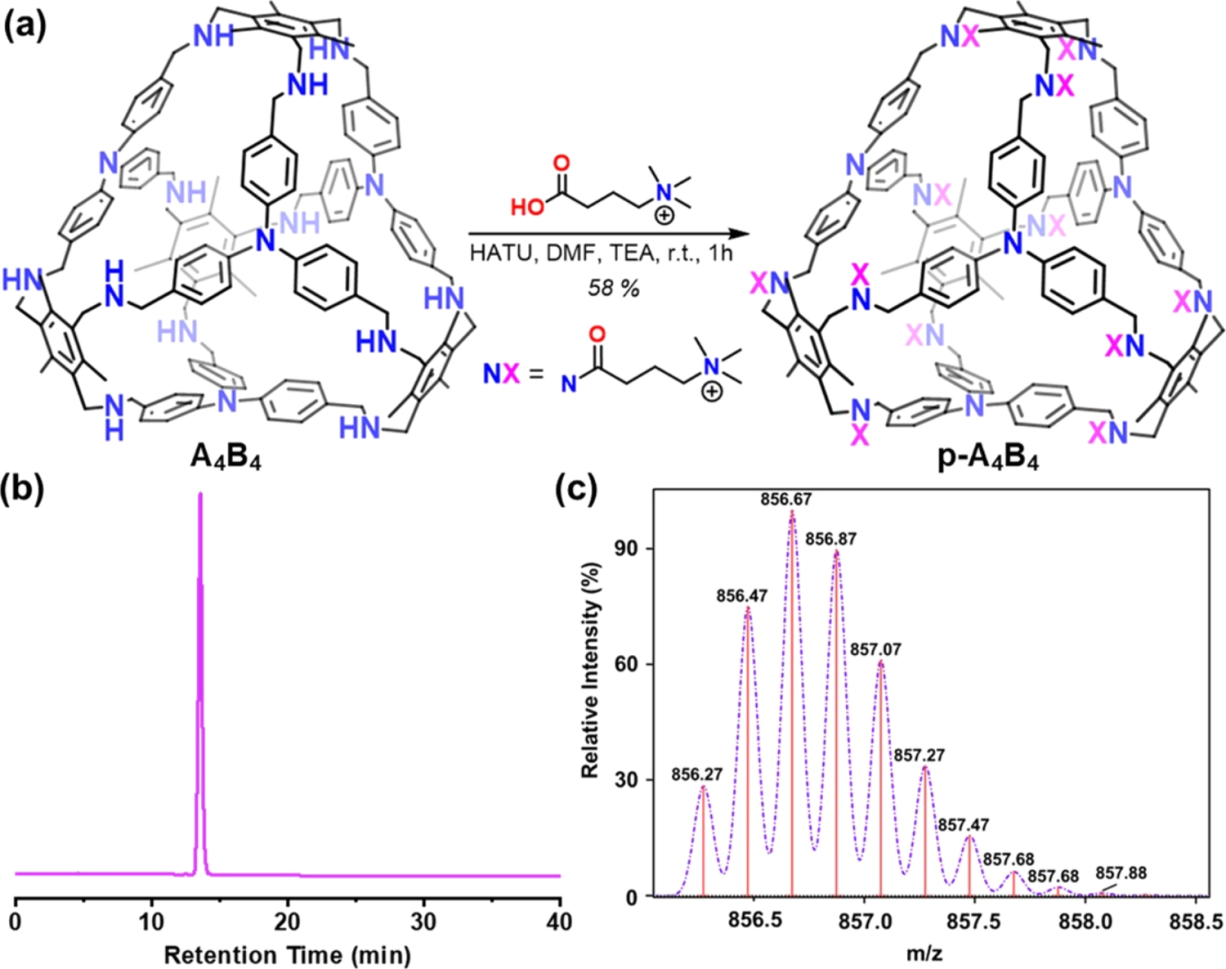
Synthesis and characterization of **p-A**_**4**_**B**_**4**_. (a) Preparation of the molecular cage. (b) HPLC chromatogram obtained for purified **p-A**_**4**_**B**_**4**_. The conditions of the analysis went from 95% of water to 95% of acetonitrile, both with 0.1% of TFA, in 40 min. (c) Predicted (purple profile) and obtained (red lines) MS spectra for **p-A**_**4**_**B**_**4**_. Spectrum shows *m*/*z* for C_216_H_311_N_28_O_12_ [M+7TFA]^5+^.

In addition to increased stability, covalent locking through imine reduction offers the advantage of forming secondary amino groups that can serve as valuable sites for binding additional functionalities through an extra postassembly modification.^43^ Until now, this strategy has exclusively demonstrated utility in the advancement of shape-persistent porous materials.^23,25^ We opted to investigate the potential use of these amino groups to address the solubility constraints of **A**_**4**_**B**_**4**_. Specifically, we chose to attach positively charged pendants, aiming to not only impart water solubility but also enhance the cage’s recognition abilities toward anionic molecules. Consequently, we synthesized a novel derivative, referred to as **p-A**_**4**_**B**_**4**_ (Figure 3).

### 2.2 Synthesis and Characterization of **p-A**_**4**_**B**_**4**_ Cage

The synthesis of **p-A**_**4**_**B**_**4**_ was accomplished through a straightforward method commonly employed in the synthesis of peptides.^44^ The process involved incubating **A**_**4**_**B**_**4**_ with an excess of the molecule 3-carboxy-*N*,*N*,*N*-trimethylpropan-1-aminium hexafluorophosphate while utilizing the coupling agent hexafluorophosphate azabenzotriazole tetramethyl uronium (HATU). This resulted in the formation of an amide bond between the secondary amino groups of **A**_**4**_**B**_**4**_ and the carboxylic group of the cationic molecule, ultimately yielding the desired product, **p-A**_**4**_**B**_**4**_. ^1^H NMR spectroscopy at room temperature displayed broad peaks in both water and DMSO, which were not useful for its characterization (Figures S12 and S13). This was anticipated due to the restricted rotation caused by the functionalization of the amino groups. ^1^H NMR spectrum in water at 90 °C leads to more defined signals, whose integrals agreed with the number of protons in **p-A**_**4**_**B**_**4**_ (Figure S14). To validate its identity and purity, conventional techniques used in biomolecule analysis, such as those for peptides, were employed. High-resolution mass spectrometry was utilized for identification (Figure 3). Reverse-phase high-performance liquid chromatography (HPLC) was employed for both purification (preparative HPLC) and purity assessment (analytical one) due to its ability to separate reaction intermediates in the formation of **p-A**_**4**_**B**_**4**_ (Figure S9).

In contrast to its precursor, **p-A**_**4**_**B**_**4**_ showed a solubility of around 2.5 mg/mL in both water and PBS buffer at neutral pH. This characteristic prompted us to investigate its recognition abilities in aqueous environments. **p-A**_**4**_**B**_**4**_ possesses a hydrophobic cavity surrounded by 12 positively charged pendants, making it a promising candidate for the recognition of hydrophobic anions. On this basis and the relevance of surfactants, we were motivated to investigate anionic surfactants as plausible guests.

### 2.3 Interaction between **p-A**_**4**_**B**_**4**_ and SDS Surfactant

**Figure 4:**
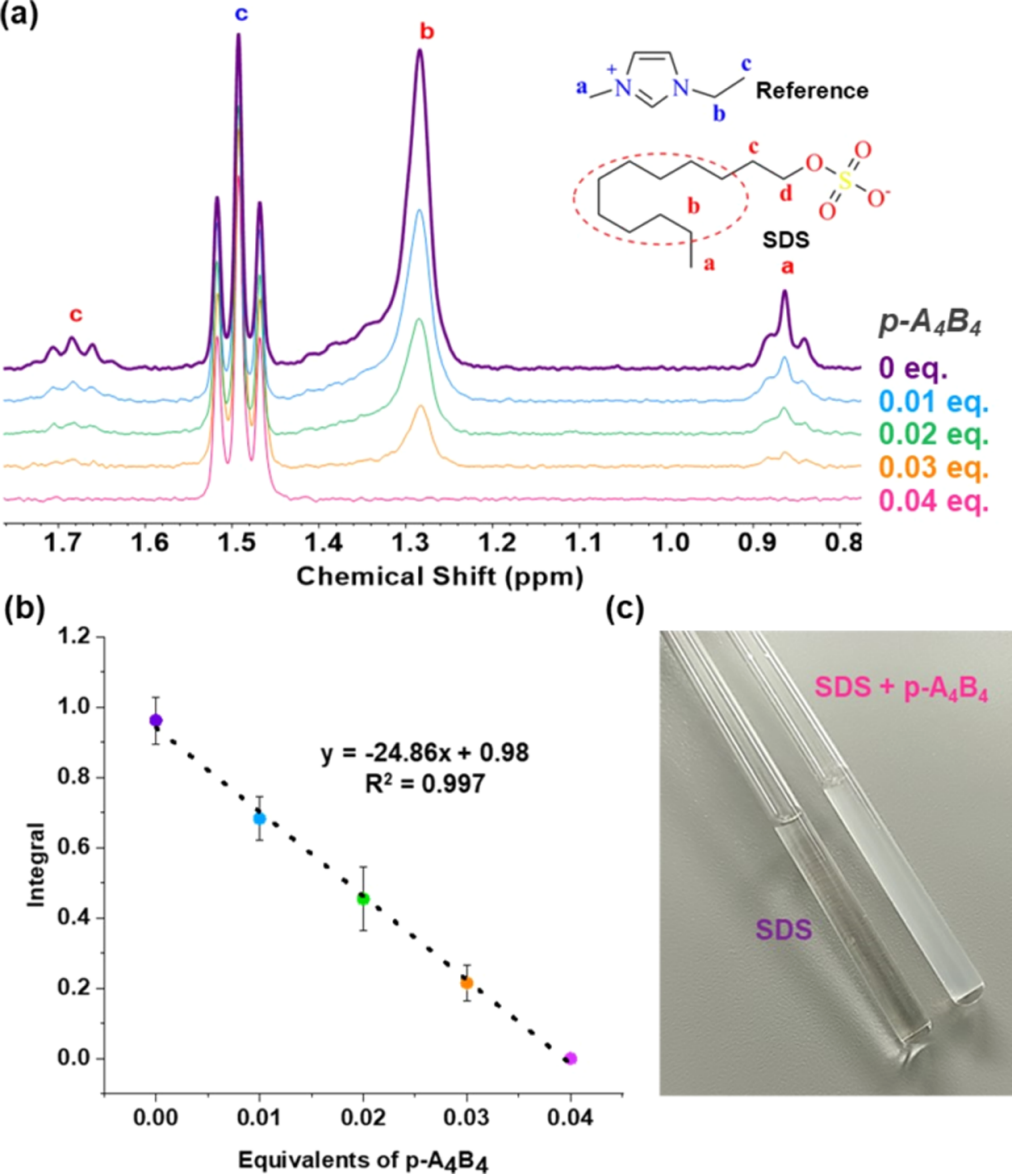
Interaction of **p-A**_**4**_**B**_**4**_ with SDS. (a) ^1^H NMR spectra for the titration of SDS (1 mM) with cage **p-A**_**4**_**B**_**4**_ (from 0 to 0.04 equivalents) in D_2_O, where the complete disappearance of the surfactant signal is observed. (b) Linear fit for the titration, where the equivalents of **p-A**_**4**_**B**_**4**_ are represented vs the surfactant’s methyl group integral. (c) Photo of the NMR tubes showing the precipitate formed after the addition of 0.04 equiv of **p-A**_**4**_**B**_**4**_ to the solution of SDS (1 mM).

A fascinating outcome emerged when an aqueous solution of sodium dodecyl sulfate (SDS, 1 mM) was titrated with **p-A**_**4**_**B**_**4**_. As depicted in Figure 4, we observed the disappearance of the SDS signal and the concurrent formation of a solid within the NMR tube. What made this observation even more intriguing was the fact that a mere 0.04 equiv of the cage sufficed to trigger the precipitation of all of the SDS molecules. To quantify the reduction in the SDS signal, we conducted a titration using 1-ethyl-3-methylimidazolium chloride as an internal standard. Remarkably, it was observed that the reduction in SDS concentration exhibited a linear relationship with the addition of the cage with a slope of approximately −24 (Figure 4b). This finding suggests that each cage, possessing a charge of +12, has the capacity to induce the precipitation of 24 anionic SDS molecules. Essentially, this indicates the precipitation ability of two surfactant molecules per positive charge within the cage. It is crucial to emphasize that the CMC of SDS is approximately 8 mM.^45^ Therefore, SDS molecules do not form micelles at the concentration employed in the titration.

To apply **p-A**_**4**_**B**_**4**_ effectively for the removal of SDS molecules in practical applications, this cage must induce precipitation even in the presence of salts. To explore this, we prepared a solution containing SDS (1 mM) in a 10 mM PBS solution using deuterated water. Upon the addition of 0.04 equiv of the cage, all of the surfactants were removed, mirroring the outcome observed in pure water (Figure S24).

To assess the specificity of this phenomenon, we conducted similar experiments involving ^1^H NMR titrations in D_2_O with hydrophobic anions distinct from the surfactant molecules. Our selection comprised negatively charged aromatic compounds with varying numbers of sulfonate groups, which included tetra(4-sulfonatophenyl)porphyrin, 8-hydroxypyrene-1,3,6-trisulfonate, hexafluorophosphate, and p-toluene sulfonate (Figures S16–S19). Remarkably, no precipitation of the guest molecules was observed even when the syringe was used in a one-to-one molar ratio.

### 2.4 Exploring the Interaction of p-A_4_B_4_ with Other Surfactants

The previous observations with hydrophobic anions suggested that precipitation is not solely attributed to electrostatic interactions; rather, the aliphatic chain of SDS plays a significant role. To explore the relationship between the aliphatic chain and precipitation phenomena, we investigated two SDS analogues with aliphatic chains of varying lengths, both of which are negatively charged sulfonate surfactants. The initial analogue comprises a sulfonate headgroup in conjunction with a 16-carbon aliphatic chain, named **16C**, exhibiting a CMC of 0.22 mM.^46^ In contrast, the second analogue possesses a shorter tail comprising only six carbons, designated as **6C**. While this analogue can serve as a cosurfactant, its abbreviated aliphatic tail restricts its ability to independently self-assemble into micelles at concentrations lower than 0.5 M.^47^

**Figure 5:**
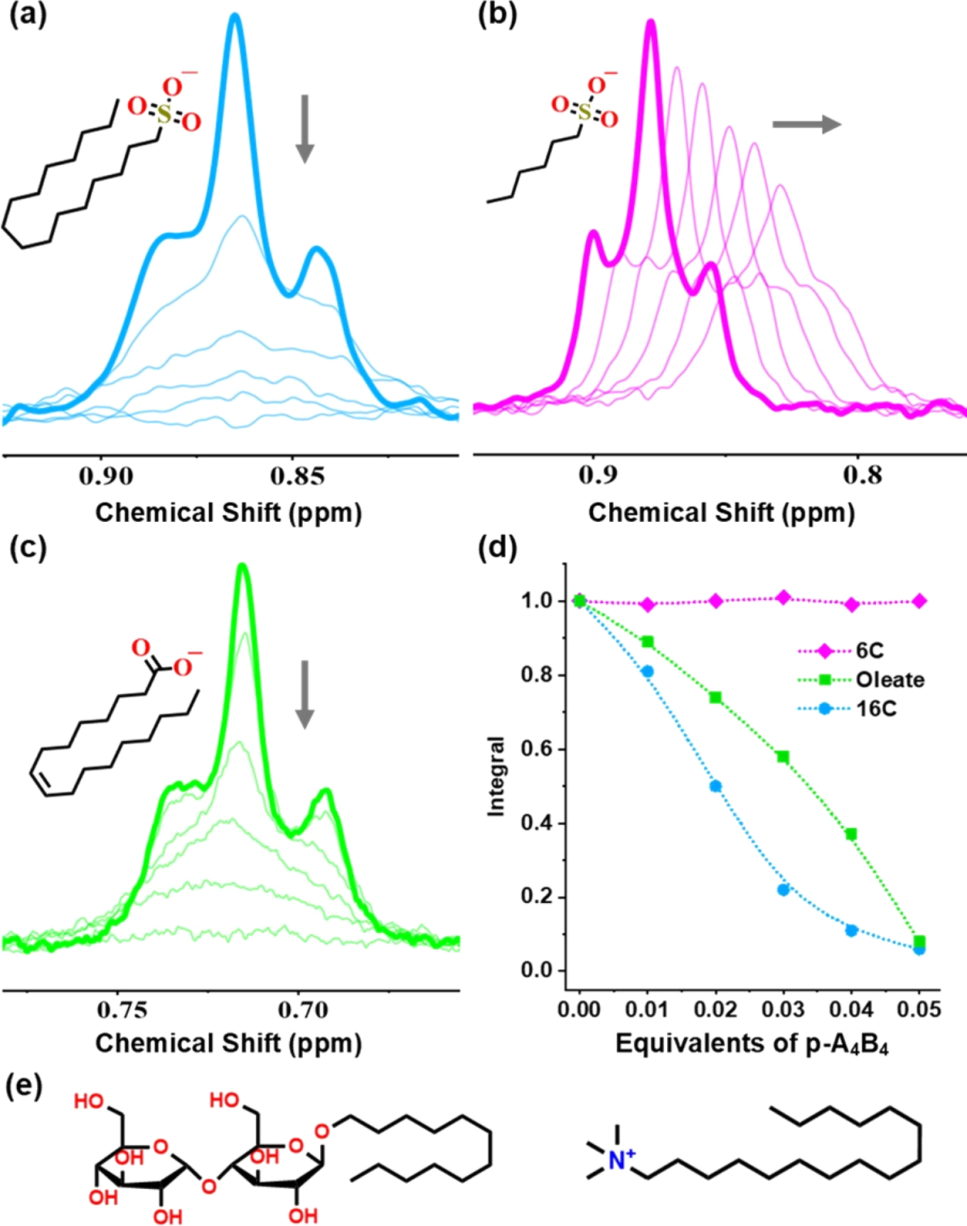
^1^H NMR spectra (300 MHz, 298 K) for the titration of **p-A**_**4**_**B**_**4**_ and three different surfactants (1 mM) with **p-A**_**4**_**B**_**4**_ in D_2_O. (a) **16C**, (b) **6C**, and (c) oleate. (d) Representation of the data obtained from the titration of the former surfactants, showing the decrease in the signal corresponding to the terminal methyl group of each one. The represented data were the result of three different titrations for each surfactant. (e) Structures of the surfactants that did not show interaction with **p-A**_**4**_**B**_**4**_: the neutral *n*-dodecyl-β-d-maltoside (left) and the positively charged CTAB (right).

When the **16C** surfactant is subjected to ^1^H NMR titration with **p-A**_**4**_**B**_**4**_, it exhibited behavior analogous to that of SDS, resulting in precipitation (Figure 5a). In this specific scenario, it was observed that to completely precipitate the surfactant, 0.05 equiv of the cage was required, slightly exceeding the 0.04 equiv needed for SDS. It is notable that the precipitation pattern exhibited by **p-A**_**4**_**B**_**4**_ displayed a more pronounced sigmoidal shape compared to that observed with SDS (Figure 5d). These effects could stem from the surfactant’s higher propensity to form micelles in comparison to SDS. Conversely, the **6C** exhibited behavior reminiscent of aromatic hydrophobic anions, as evidenced by ^1^H NMR spectroscopy, which revealed interactions but no precipitation (Figure 5b). During the titration with **p-A**_**4**_**B**_**4**_, a discernible trend was evident in the aliphatic signals of **6C**; they exhibited a consistent upfield shift and broadening, maintaining a constant integral.

At this point, we decided to explore how general the interaction of **p-A**_**4**_**B**_**4**_ was with surfactants. Consequently, we undertook an evaluation of this cage’s interaction with sodium oleate. In contrast to previously examined surfactants, oleate features a carboxylate moiety as its polar headgroup, while its aliphatic tail comprises 18 carbon atoms and a single unsaturation. Despite its distinct chemical structure compared to SDS, the outcomes closely resembled those observed with prior surfactants, demonstrating complete precipitation upon the addition of 0.05 equiv of **p-A**_**4**_**B**_**4**_ (Figure 5c). Subsequently, we shifted our focus to positively charged surfactants, particularly cetyltrimethylammonium bromide (CTAB), which has a 16-carbon aliphatic chain and a CMC of 1.0 mM.^33^ Remarkably, **p-A**_**4**_**B**_**4**_ did not exhibit any discernible interaction with CTAB by NMR (Figure S30). Finally, we evaluated a neutral surfactant, n-dodecyl-β-D-maltoside, featuring a 12-carbon aliphatic tail and a neutral polar head consisting of a disaccharide. However, as observed through ^1^H NMR titration, there was no interaction or precipitation, mirroring the results obtained with CTAB (Figure S32).

These experiments underscore that the interaction of **p-A**_**4**_**B**_**4**_ with other molecules is predominantly driven by the electrostatic attraction, as it only exhibited an interaction with anionic molecules. However, the precipitation phenomenon is specific to anionic surfactants. It is noteworthy that this precipitation occurs at concentrations significantly lower than the surfactant CMC.

### 2.5 Understanding Cage–Surfactant Interaction

To gain a microscopic understanding of cage–surfactant interactions, we performed molecular dynamics (MD) simulations of **p-A**_**4**_**B**_**4**_ in the presence of different surfactants in an aqueous solution. We specifically examined interactions with SDS, CTAB, and **6C** to evaluate effects related to the ionic character and aliphatic tail length. Two scenarios were considered. In the first, systems featured a single **p-A**_**4**_**B**_**4**_ in the presence of 48 surfactants to evaluate the nature of **p-A**_**4**_**B**_**4**_/surfactant interactions. In the second, the systems featured three **p-A**_**4**_**B**_**4**_ and surfactants to assess the propensity for precipitation via aggregation of multiple **p-A**_**4**_**B**_**4**_.

**Figure 6:**
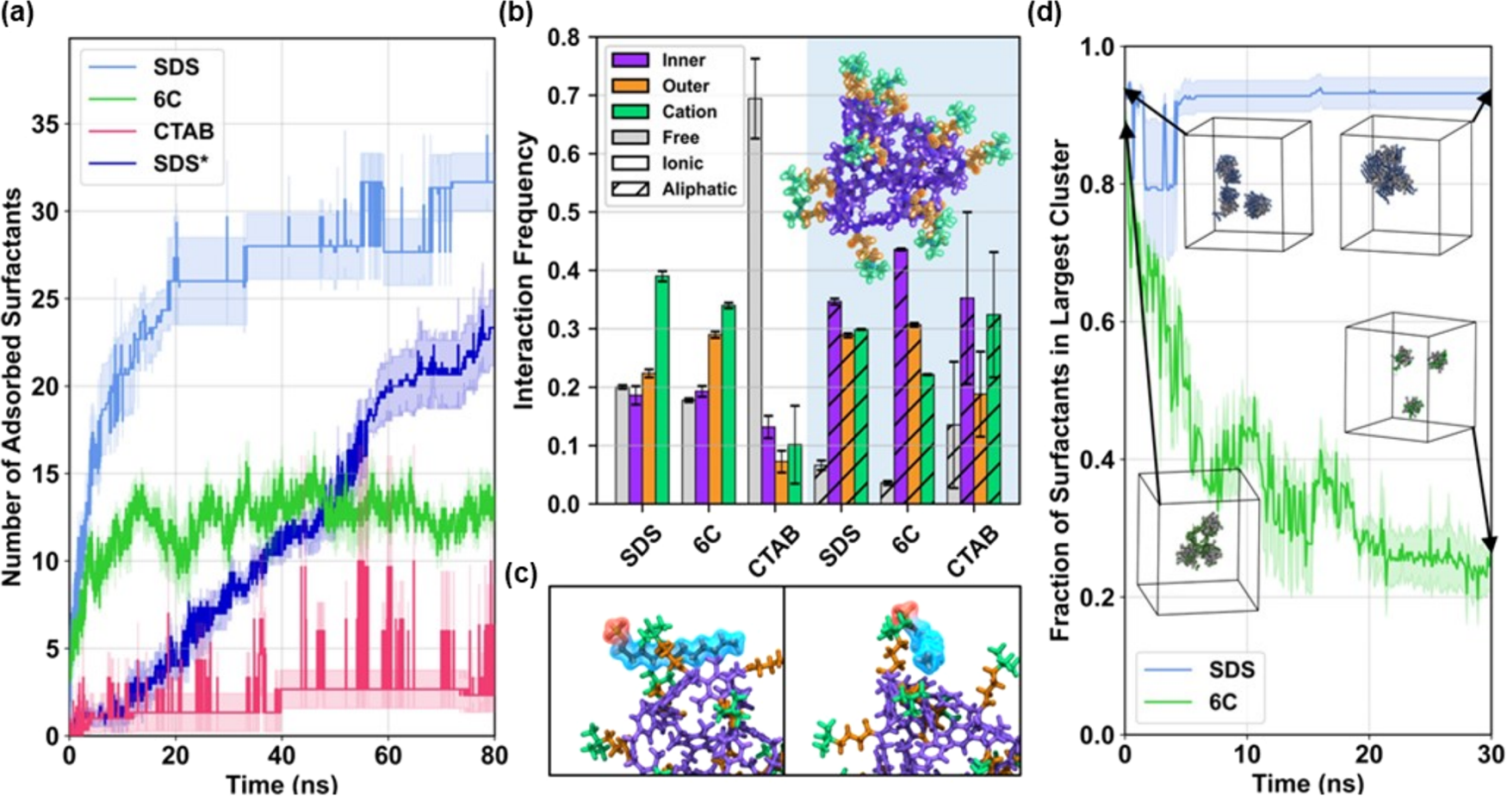
MD simulations of **p-A**_**4**_**B**_**4**_ and the surfactants. (a) Number of surfactants in the vicinity of **p-A**_**4**_**B**_**4**_ as a function of time. SDS* represents the gradual introduction of SDS into the cell to emulate the dilution conditions. (b) The relative frequency or proportion of charged (ionic, left) and neutral (aliphatic, right) groups of surfactant molecules interacting with inner, outer, or cationic regions of **p-A**_**4**_**B**_**4**_. Surfactant interactions with **p-A**_**4**_**B**_**4**_ are defined if any atoms between the two groups are within 3.0 Å. Surfactants that are not in the vicinity of **p-A**_**4**_**B**_**4**_ are labeled “free”. The inset shows the decomposition of regions defined for **p-A**_**4**_**B**_**4**_, with the inner, outer, and cationic regions highlighted in purple, orange, and green, respectively. (c) Representative simulation snapshot of SDS interacting with **p-A**_**4**_**B**_**4**_. The anionic headgroup is shown in red, while the aliphatic group is shown in blue. (d) Fraction of surfactant molecules assigned to the largest aggregate for simulations with multiple **p-A**_**4**_**B**_**4**_ units. Insets show **p-A**_**4**_**B**_**4**_ molecules and their attached surfactants.

Figure 5a demonstrates that **p-A**_**4**_**B**_**4**_ exhibits a significantly higher affinity for SDS compared to that of CTAB or **6C**. Initially, we conducted simulations with randomly distributed surfactants in a simulation cell, monitored their distances from **p-A**_**4**_**B**_**4**_ over time, and employed clustering analysis to evaluate binding. These simulations showed that approximately 25–31 SDS molecules promptly bind to **p-A**_**4**_**B**_**4**_, while the number of bound CTAB and **6C** surfactants plateaus at lower values. The increased binding of **6C** compared to that of CTAB and the overall number of bound SDS align well with experimental findings. Because these simulations featured surfactant concentrations above the CMC, surfactants often formed small aggregates that would subsequently adsorb to **p-A**_**4**_**B**_**4**_, as evident in the discrete jumps in Figure 6a. To mimic interactions under more diluted conditions, additional simulations gradually introduced SDS to the cell, yet SDS continued to bind effectively to **p-A**_**4**_**B**_**4**_ (SDS* in Figure 6a). These findings suggest that the simulations accurately capture the essential physics of surfactant/**p-A**_**4**_**B**_**4**_ systems, such as the relative affinity of **p-A**_**4**_**B**_**4**_ toward anionic surfactants and the formation of aggregates under diluted conditions.

The enhanced binding of anionic surfactants led to the hypothesis that surfactants specifically interact with cationic portions of **p-A**_**4**_**B**_**4**_. To explore this, we monitored how often the ionic and aliphatic groups of adsorbed surfactants were interacting with the ″inner″ (derived from **A**_**4**_**B**_**4**_), “outer” (the neutral part of the pendants), and “cationic” (the positively charged quaternary ammonium) regions of **p-A**_**4**_**B**_**4**_. To characterize the relative proportion of such interactions, we define “interaction frequency” as the normalized number of occurrences where any atoms from the aliphatic or ionic portions of adsorbed surfactants **p-A**_**4**_**B**_**4**_ were within a 3.0 Å radial cutoff of any atoms from a specified region of **p-A**_**4**_**B**_**4**_. Figure 6b reveals that the ionic group of anionic surfactants indeed preferentially interacts with the cationic regions of **p-A**_**4**_**B**_**4**_. However, there are also a substantial number of interactions between aliphatic groups and all regions of **p-A**_**4**_**B**_**4**_. While the behavior is relatively similar between SDS and **C6**, interactions between CTAB and **p-A**_**4**_**B**_**4**_ are less specific, particularly for its ionic group. Across all surfactants, the aliphatic tails exhibit some preference toward the inner region of **p-A**_**4**_**B**_**4**_. Taken together, these results suggest that both electrostatic and dispersion interactions underlie the functionality of **p-A**_**4**_**B**_**4**_, as selective binding is principally driven by **p-A**_**4**_**B**_**4**_’s cationic functionalization, while the hydrophobic core region offers additional stabilizing interactions with aliphatic groups. In summary, these results elucidate why a single cage can precipitate 24 molecules of SDS. The interaction with surfactants takes place on the exterior of the cage rather than through encapsulation within the cage cavity. As a result, the cage can accumulate a substantial number of surfactant molecules on its surface.

We next examined how the enhanced binding of anionic surfactants might manifest in the observed precipitation. For this, single **p-A**_**4**_**B**_**4**_/surfactant complexes from previous simulations involving SDS and **C6** were initialized in proximity and surrounded by additional surfactants to seed an aggregate of multiple **p-A**_**4**_**B**_**4**_; CTAB was not considered due to its prior minimal association with a single **p-A**_**4**_**B**_**4**_. Simulations were then run to assess whether such aggregates would remain stable. Figure 5d illustrates contrasting behavior between surfactants in terms of the multicage assembly of **p-A**_**4**_**B**_**4**_. Although both SDS and **6C** initially display large aggregates with multiple **p-A**_**4**_**B**_**4**_ and most of the surfactants by virtue of the initialization procedure, such aggregates gradually dissipate for **6C** while they remain stable in the presence of SDS. Importantly, this observation facilitates comprehension regarding the exclusive ability of surfactants to enable multicage assembly. We hypothesize that this assembly process precedes subsequent precipitation events.

## 3 Experimental Section

### 3.1 Synthesis of A_4_B_4_ Cage

A solution of tris(4-formylphenyl)amine **A** (100 mg, 0.303 mmol, 4.0 equiv) and (2,4,6-trimethylbenzene-1,3,5-triyl)trimethanamine **B** (65 mg, 0.602 mmol, 4.1 equiv) dissolved in CHCl_3_ (80 mL) was heated at 60 °C for 4 days (based on the protocol previously described by Cooper’s group^[1]^). The reaction was allowed to cool to room temperature, and 80 mL of MeOH and 30 mg of NaBH_4_ were added. The solution was left stirring at room temperature overnight. Then, the solvent was removed under vacuo and 8 mL of 1 M NaOH was added. The obtained suspension was centrifuged, and the solid was washed 3 times with water. The resulting solid was dried, and the final compound was obtained as a pale-yellow solid in a quantitative yield.

### 3.2 Synthesis of p-A_4_B_4_

3-Carboxy-*N,N,N*-trimethylpropan-1-aminium hexafluorophosphate (75 mg, 15 equiv), HATU (146 mg, 15 equiv), and triethylamine (320 μL, 30 equiv) were dissolved in 5 mL of DMF and stirred at room temperature until the apparition of a yellowish color (around 5 min). Then, **A**_**4**_**B**_**4**_ was added to the mixture (50 mg, 1 equiv). The resulting yellow solution was left stirring at room temperature for 1 h. Purification was done by HPLC, and 52 mg of product was obtained as a white solid (**p-A**_**4**_**B**_**4**_, 58%).

### 3.3 Purification and Characterization Techniques

HPLC purification was carried out using a Fortis C18 semipreparative column (5 μm, size: 250 × 10 mm^2^) with phase A/phase B gradients (phase A: H_2_O with 0.1% trifluoroacetic acid; phase B: acetonitrile with 0.1% trifluoroacetic acid). Proton nuclear magnetic resonance (^1^H NMR), carbon nuclear magnetic resonance (^13^C NMR), and fluorine nuclear magnetic resonance (^19^F-NMR) spectra were measured on a Bruker AVANCE III HD 300 nuclear magnetic resonance spectrometer or a Bruker AVANCE III HD 500 nuclear magnetic resonance spectrometer and were referenced relating to residual proton resonances in CDCl_3_ (at δ 7.24 ppm), D_2_O (at δ 4.79 ppm), MeOD (at δ 3.31 ppm), and CD_3_SO (at δ 2.50 ppm). Carbons are referenced relating to residual carbon resonances in MeOD (at δ 77.23 ppm). ^1^H NMR splitting patterns are assigned as singlet (s), doublet (d), triplet (t), or quartet (q). Splitting patterns that could not be readily interpreted are designated as multiplet (m). All chemical shift (δ) values are given in parts per million. All coupling constants are quoted in Hz. All ^13^C and ^19^F spectra are proton-decoupled unless otherwise stated. Mass spectra were recorded in positive mode using an electrospray ionization technique (ESI) in an LC-Q-q-TOF Applied Biosystems QStar Elite mass spectrometer located at the Research Support Services, SAI (Servizos de Apoio á Investigación), of the University of A Coruña. The predicted mass spectra were calculated using mMass Software, version 5.5.0.

### 3.4 MD Simulations

All MD simulations were conducted using the LAMMPS simulation package (stable release 23 Jun 2022). Force field parameters for the inner region of **p-A**_**4**_**B**_**4**_ were taken from the all-atom optimized potentials for liquid simulations (OPLS-AA) force field, while parameters for the outer and cationic regions of **p-A**_**4**_**B**_**4**_ were taken from the Canongia Lopes & Padua (CL&P) force field. Parameters for one atom type, an aromatic carbon bonded to a tertiary amine, were not present in either force field. This atom type was assigned a partial charge to maintain a +12 formal charge on **p-A**_**4**_**B**_**4**_, and OPLS-AA parameters for a standard aromatic carbon were used for all other interactions. Force field parameters for the neutral portions of surfactants were taken from the OPLS-AA force field, while parameters for the charged head groups were taken from the CL&P force field. All parameters for counterions were obtained from the CL&P force field. Water was modeled using the rigid TIP4P model. Real-space nonbonded interactions were truncated at 12.0 Å. Long-range electrostatics were handled using the particle–particle–particle–mesh Ewald summation method with a convergence accuracy of 10^–5^.

Cubic simulation cells were used for all simulations, with periodic boundary conditions applied in every dimension. Simulations containing a single **p-A**_**4**_**B**_**4**_ molecule used a box length of 90 Å, while simulations containing three **p-A**_**4**_**B**_**4**_ molecules used a box length of 130 Å. Simulation cells were initially prepared by inserting the desired molecules into their appropriate counterions. Sodium cations were used as the counterions for anionic surfactants, bromine anions were used as the counterions for CTAB, and 12 trifluoroacetic acid molecules were used as counterions for **p-A**_**4**_**B**_**4**_. All systems were solvated with randomly inserted water molecules to achieve a density of 1000 kg/m^3^. Water molecules were not included if they could not be placed at a distance of at least 1.3 Å from other molecules in the system.

All systems underwent 10,000 steps of energy minimization. Systems were initially relaxed for 50 ps at 300 K using a Nosé–Hoover thermostat with a damping constant of 100 fs. The systems were then subject to a brief equilibration for 100 ps at 300 K and 1 bar by using a Nosé–Hoover thermostat and barostat. Given that rigid TIP4P water required separate treatment of dynamics, *NPT* equilibration coupled only water molecules to the Nosé–Hoover barostat, while all remaining molecules were coupled to a Nosé–Hoover thermostat. All production-run simulations used the same conditions as those used for the *NPT* equilibration. Equations of motion were evolved using a velocity-Verlet integration scheme with a 1 fs time step.

## 4 Conclusions

We have achieved precise modulation of the solubility and host–guest properties in molecular cages originating from imine self-assembly. This accomplishment is attributed to a dual-step postassembly refinement: first, a covalent locking mechanism involving the reduction of imine bonds and, second, the harnessing of reactivity in the generated secondary amino groups to bind positively charged pendant groups. This modification not only rendered the cage water-soluble but also endowed it with a tailored affinity for anionic surfactant that enables its complete removal.

## Data Availability

Simulation files needed to reproduce the MD simulations are available at https://github.com/webbtheosim/surfactant-containers.

## Supporting Information Available

Detailed experimental procedures; characterization (NMR spectroscopy, high-performance liquid chromatography [HPLC], HRMS spectrometry); and computational procedures (PDF)
