# Supplementary material for: Engineering a Surfactant Trap via Postassembly Modification of an Imine Cage
Source: Chem Mater. 2024 Sep 4;36(18):8920–8. doi: 10.1021/acs.chemmater.4c01808 (PMC11428146; doi:10.1021/acs.chemmater.4c01808)
Supplement: Supplementary file 1 — cm4c01808_si_001.pdf [file cm4c01808_si_001.pdf]

Supplemental Information

Engineering a Surfactant Trap via Post-Assembly Modification of an Imine Cage

María Pérez-Ferreiro,[a] Quinn M. Gallagher,[b] Andrea B. León,[a] Michael A. Webb,*,[b] Alejandro Criado,*,[a] and Jesús Mosquera*,[a]

[a]Universidade da Coruña, CICA−Centro Interdisciplinar de Química e Bioloxía, Rúa as Carballeiras, 15071 A Coruña, Spain

[b]Department of Chemical and Biological Engineering, Princeton University, Princeton, NJ 08544 USA

Table of Contents

1. Materials and general methods 1

2. Synthesis and characterization 2

2.1. Building block B 2

2.2. Molecular cage A_4B_4 5

2.3. Functionalized molecular cage p-A_4B_4 10

3. Interaction of p-A_4B_4 with hydrophobic anions 15

3.1. Tetra(4-sulfonatophenyl)porphyrin 15

3.2. 8-hydroxypyrene-1,3,6-trisulfonate 16

3.3. p-toluenesulfonate 17

4. Interaction of p-A_4B_4 with surfactants 18

4.1. 6C surfactant 18

4.2. SDS surfactant 19

4.3. 16C surfactant 21

4.4. Sodium oleate 23

4.5. CTAB 25

4.6. n-Dodecyl-β-D-maltoside 26

5. Computational methods: MD simulations 27

6. References 28

1. Materials and general methods

All reagents (**Table S1**) and solvents (**Table S2**) employed were commercially available and used as supplied without further purification unless stated otherwise.

Table S1. *Reagents used for the synthesis.*

REAGENTS	PROVIDER
5'-(4-formylphenyl)-[1,1':3',1''-terphenyl]-4,4''-dicarbaldehyde (**A**)	*Panreac*
Tris(2-aminoethyl)amine (**B**)	*Aldrich*
3-carboxy-N,N,N-trimethylpropan-1-aminium chloride (**F**)	*BLD Pharmatech*
O-(7-Azabenzotriazol-1-yl)-N,N,N',N'-tetramethyluronium hexafluorophosphate (HATU)	*Apollo Scientific*
1,3,5-tris(bromomethyl)-2,4,6-trimethylbenzene	*Aldrich*
Potassium phthalimide	*ChemoSapiens*
Hydrazine	*Aldrich*
4,4',4''-nitrilotribenzaldehyde (**E**)	*BLD Pharmatech*
18-crown6	*Aldrich*
$NaBH_4$	*Aldrich*
Na_2SO_4	*Fluorochem*
KOH	*Panreac*
NaOH	*Panreac*
Triethylamine	*Fisher Chemical*
Trifluoroacetic acid (TFA)	*TCI*

Table S2. *Solvents used for the synthesis.*

SOLVENT	PROVIDER
Acetonitrile (CH_3CN), HPLC grade	*Panreac*
Chloroform ($CHCl_3$)	*Fisher Scientific*
Dichloromethane (CH_2Cl_2)	*Fisher Scientific*
Dimethylformamide (DMF)	*Fisher Scientific*
Ethanol (EtOH)	*Fisher Scientific*
Methanol (MeOH)	*Fisher Scientific*
Toluene	*Fisher Scientific*
Water	-----
Water HPLC grade	*Fisher Scientific*
Acetonitrile HPLC grade	*Fisher Scientific*
Deuterated chloroform	*Deutero GmbH*
Deuterated methanol	*Euriso-top*
Deuterated DMSO	*Deutero GmbH*
Deuterated water	*Deutero GmbH*

HPLC purification was carried out using a Fortis C18 semipreparative column (5 µm, size: 250×10 mm) with Phase A/Phase B gradients (Phase A: H_2O with 0.1% trifluoroacetic acid; Phase B: Acetonitrile with 0.1% trifluoroacetic acid). Proton nuclear magnetic resonance (^{1}H-NMR), Carbon nuclear magnetic resonance (^{13}C-NMR) and Fluorine nuclear magnetic resonance (^{19}F-NMR) spectra were measured on Bruker AVANCE III HD 300 Nuclear Magnetic Resonance spectrometer or a Bruker AVANCE III HD 500 Nuclear Magnetic Resonance spectrometer and were referenced relating to residual proton resonances in $CDCl_3$ (at δ 7.24 ppm), D_2O (at δ 4.79 ppm), MeOD (at δ 3.31 ppm), and CD_3SO (at δ 2.50 ppm). Carbons are referenced relating to residual carbon resonances in MeOD (at δ 77.23 ppm). ^{1}H-NMR splitting patterns are assigned as singlet (s), doublet (d), triplet (t) or quartet (q). Splitting patterns that could not be readily interpreted are designated as multiplet (m). All chemical shift (δ) values are given in parts per million. All coupling constants are quoted in Hz. All ^{13}C and ^{19}F spectra are proton decoupled unless otherwise stated. Mass spectra were recorded in positive mode using an electrospray ionization technique (ESI) in a LC-Q-q-TOF Applied Biosystems QStar Elite mass spectrometer located at the Research Support Services, SAI (Servizos de Apoio á Investigación) of the University of A Coruña. The predicted mass spectra were calculated using mMass Software, version 5.5.0.

2. Synthesis and characterization

2.1. Building block B

The synthesis of (2,4,6-Trimethylbenzene-1,3,5-triyl)trimethanamine, **B**, was carried out in two steps based on protocol described in literature.[1]

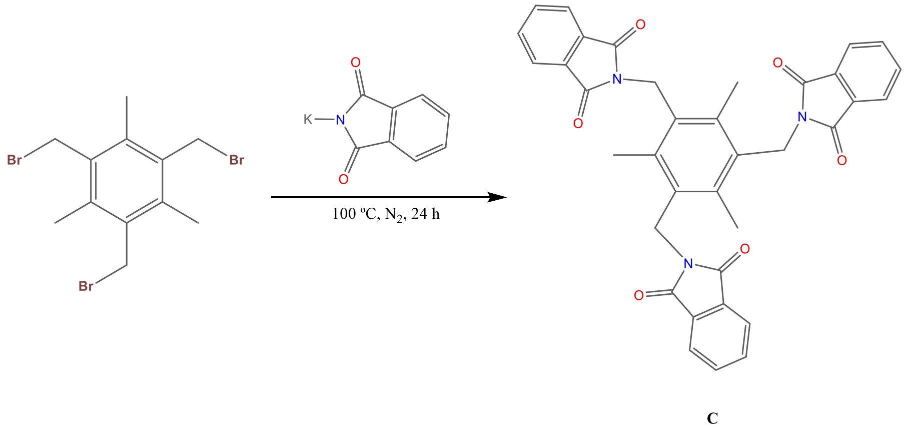

Scheme S1. *Synthesis of compound **C**, precursor of building block **B**.*

To a solution of 1,3,5-tris(bromomethyl)-2,4,6- trimethylbenzene (1.9919 g, 5.01 mmol, 1.0 eq., white powder) and 18-crown6 (0.3995 g, 1.5 mmol, 0.3 eq., translucent crystals) in toluene (64 mL, colourless liquid) was added potassium phthalamide (3.3361 g, 18.05 mmol, 3.6 eq., yellowish powder). The mixture (yellowish and dense mixture) was heated at 100 °C under N_2 for 24 h. After around 20 min the aspect of the solution changed from the yellowish and dense mixture to the formation of a large amount of white solid, which difficulted the stirring. After 24 h, the mixture was allowed to cool to room temperature. The mixture was concentrated in vacuo and the resulting solid suspended in water (40 mL) and collected by filtration. The resulting solid (white solid) was further washed with water (2 × 40 mL) and MeOH (40 mL) before being dried in vacuo to afford the desired product (**C**) as a white solid which was used without further purification (3.10 g).

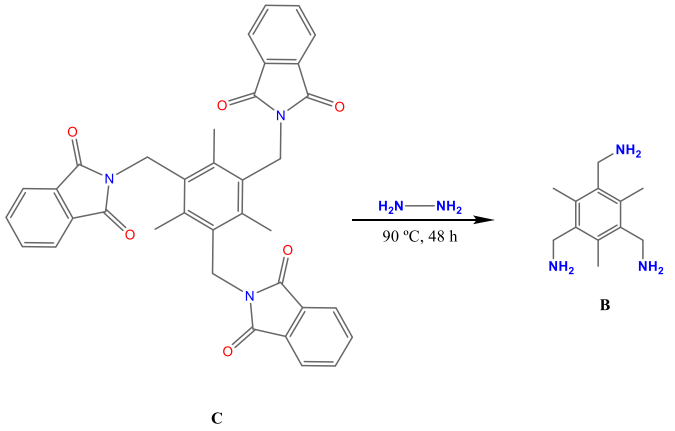

Scheme S2. *Synthesis of building block B.*

To a suspension of **C** (2.70 g, 4.52 mmol, 1.0 eq.) in a mixture of toluene (35 mL) and EtOH (75 mL) was added hydrazine hydrate in a single portion (1.6 mL, 50 wt% solution in water, 27 mmol, 6.0 eq.). The resulting mixture was heated at 90 °C for 44 h, at which point a large amount of solid had precipitated, before being allowed to cool to room temperature. The reaction mixture was concentrated in vacuo (not to dryness) and partitioned between an aqueous KOH solution (17 mL, 40 wt%) and $CHCl_3$ (44 mL). The aqueous layer was further extracted with $CHCl_3$ (2 × 23 mL) before the combined organic layers were dried over Na_2SO_4 and concentrated in vacuo to afford the desired triamine **B** as a pale-yellow solid (0.8774 g, 93 %).

^{1}H-NMR (300 MHz, CD_3OD): δ 3.89 (s, 6H), 2.43 (s, 9H) ppm. **^{13}C-NMR** (75 MHz, CD_3OD): δ 138.16, 135.26, 40.66, 15.73 ppm. Data in accordance with literature values.[1]

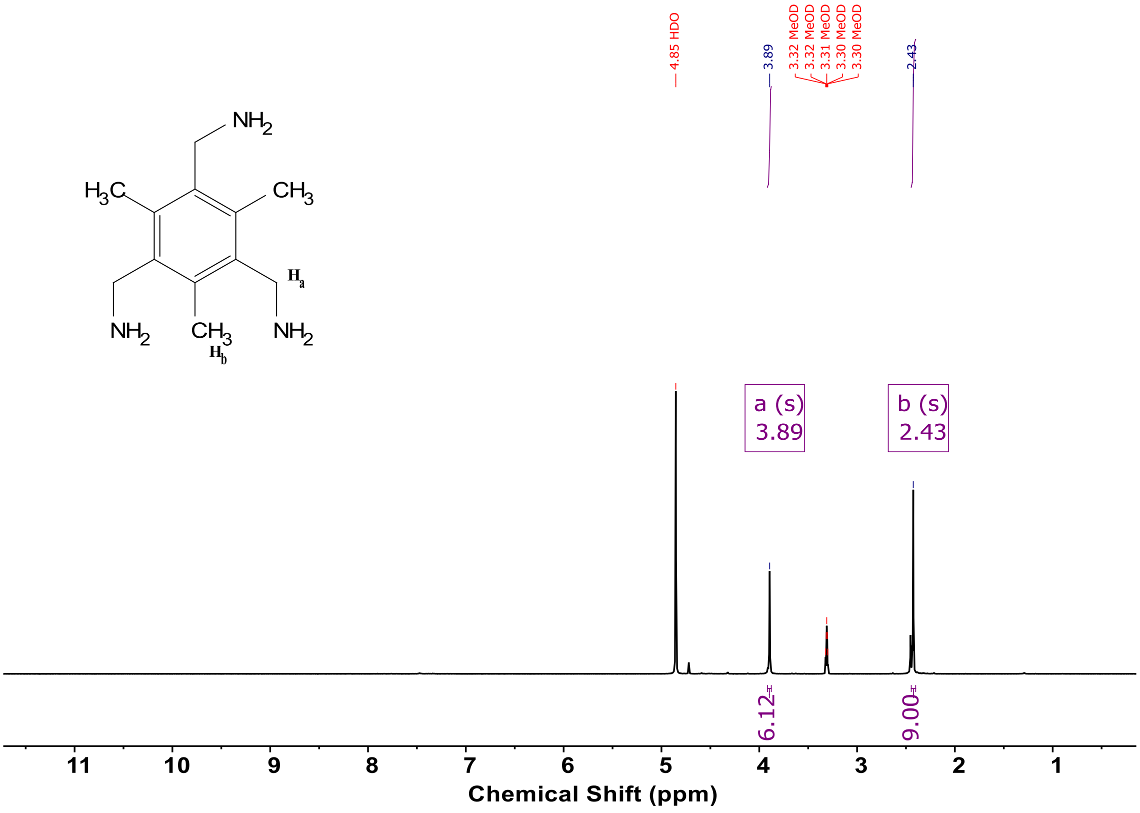

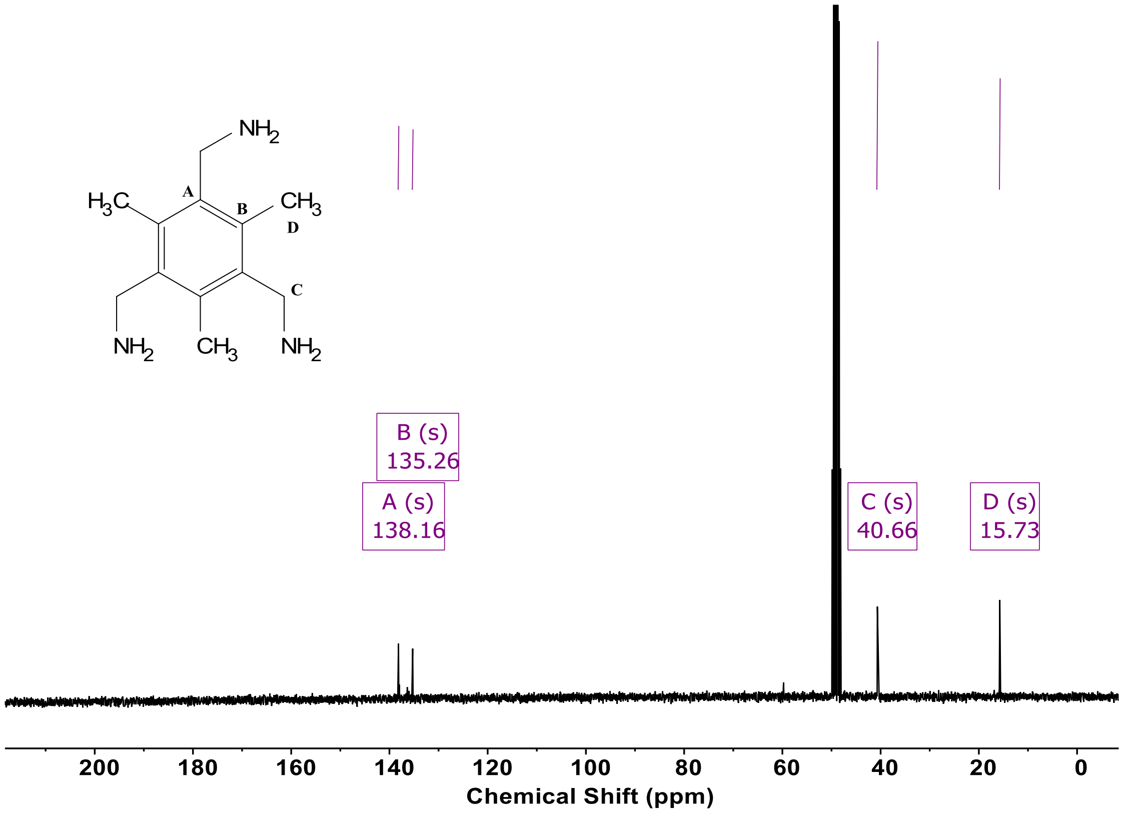

Figure S1. *^{1}H-NMR (300 MHz) (above) and ^{13}C-NMR (75 MHz) (below) spectra recorded for building block* ***B*** *in MeOD and at room temperature.*

2.2. Molecular cage A_4B_4

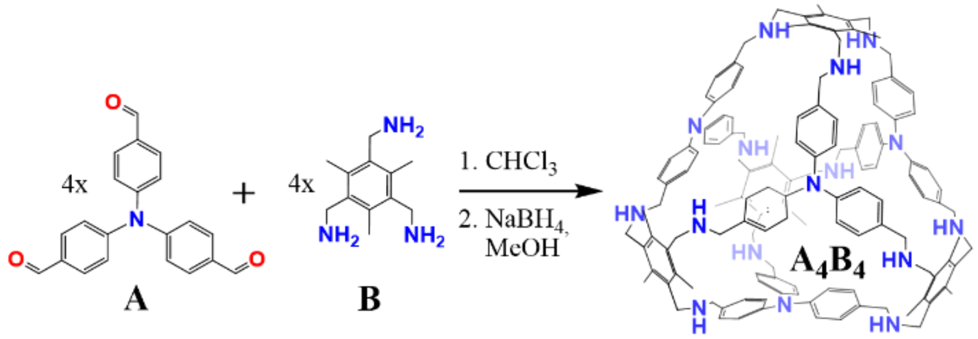

Scheme S3. *Synthetic procedure followed for the synthesis of the molecular cage* ***A_4B_4.***

A solution of tris(4-formylphenyl)amine **A** (100 mg, 0.303 mmol, 4.0 eq.) and (2,4,6-trimethylbenzene-1,3,5-triyl)trimethanamine **B** (65 mg, 0.602 mmol, 4.1 eq.) dissolved in $CHCl_3$ (80 mL) was heated at 60 °C for 4 days (based on the protocol previously described by Cooper's group[1]). Reaction was allowed to cool to room temperature and 80 mL of MeOH and 30 mg of $NaBH_4$ were added. Solution was left stirring at room temperature overnight. Then, solvent was removed under vacuo and 8 mL of NaOH 1M were added. The obtained suspension was centrifuged, and the solid was washed three times with water. The resulting solid was dried, and the final compound was obtained as a pale-yellow solid, in quantitative yield.

To improve the amide formation reaction (see **section 2.3**), the final product was passed through MPLC, using Phase A/Phase B* gradients, from 95 % to 5 % of A, with High-Performance BGB Aquarius C18AQ Flash Cartridges (100Å, Spherical, 15µm), in 30 minutes. The final product was obtained with trifluoroacetic acid as counterion, improving its solubility in DMF.

*Phase A: H_2O with 0.1% trifluoroacetic acid; Phase B: Acetonitrile with 0.1% trifluoroacetic acid

HRMS(ESI+): *m/z* calc. for $C_{132}H_{144}N_{16}$ $[M+H]^+$: 1955.19. Found: 1955.19. **^{1}H-NMR** (400 MHz, DMSO-*d*$_6$) δ 9.06 (s, 1H), 7.34 (d, *J* = 8.1 Hz, 3H), 6.80 (d, *J* = 8.2 Hz, 3H), 4.48 (s, 2H), 4.09 (s, 3H), 1.23 (s, 3H) ppm.

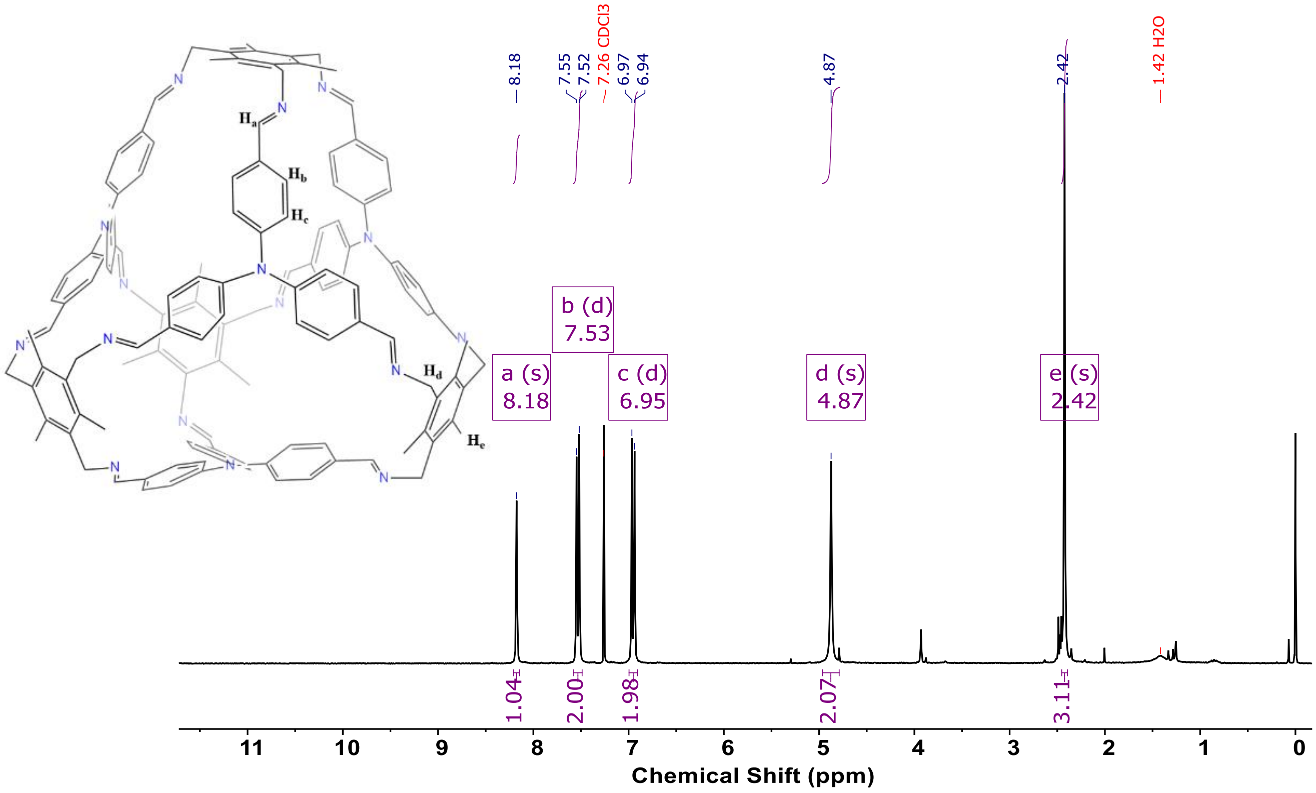

Figure S2. *^{1}H-NMR (400 MHz) characterization in $CDCl_3$ at room temperature of the self-assembled product, precursor of the reduced* ***A_4B_4*** *molecular cage.*

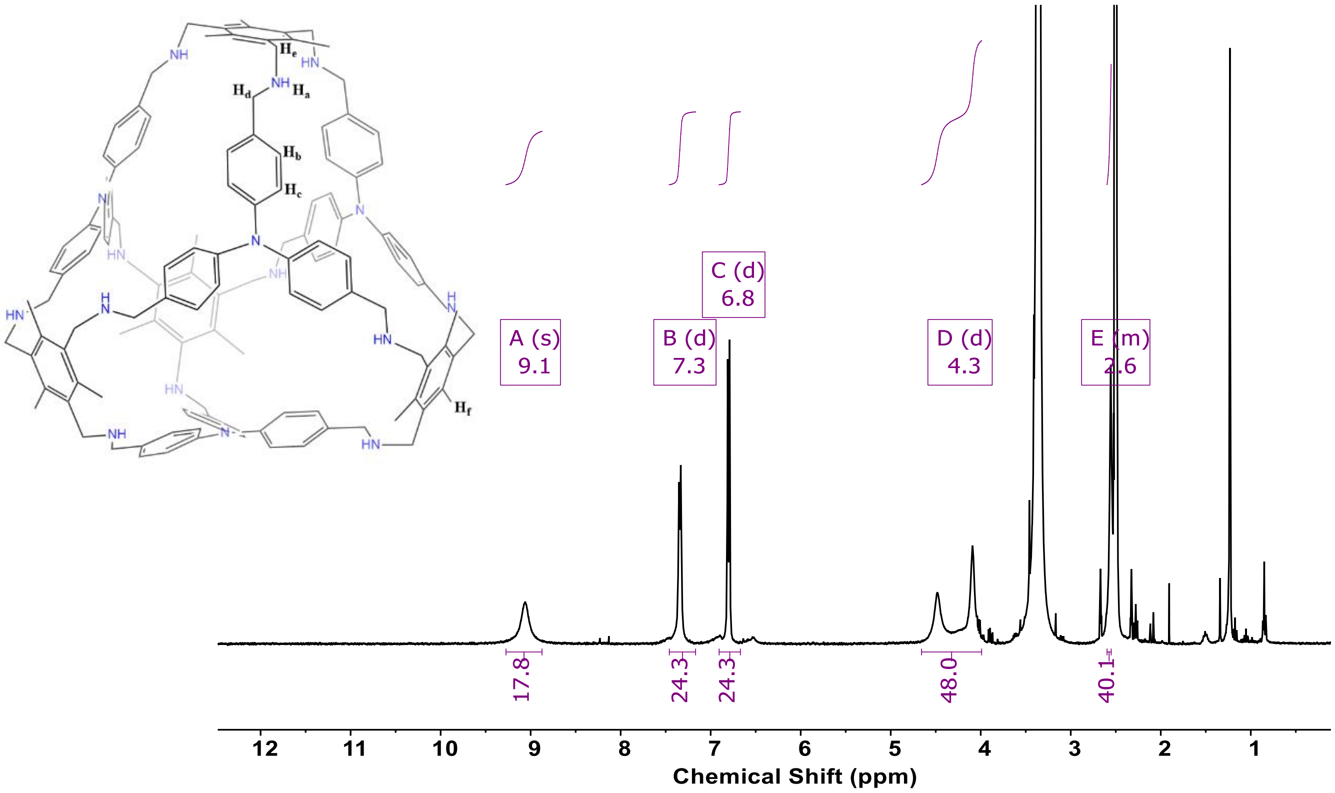

Figure S3. *^{1}H-NMR (500 MHz, 298 K) characterization of the purified reduced molecular cage* ***A_4B_4*** *in DMSO.*

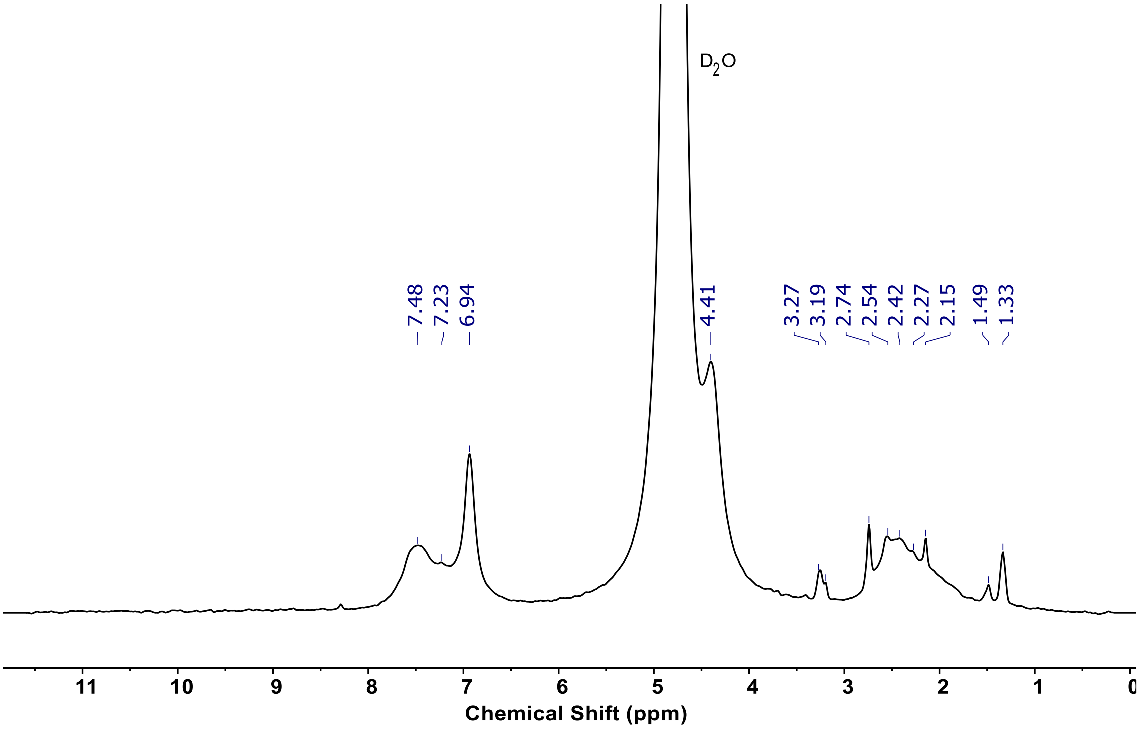

Figure S4. *^{1}H-NMR (500 MHz, 298 K) characterization in D_2O of the purified reduced molecular cage,* ***A_4B_4***.

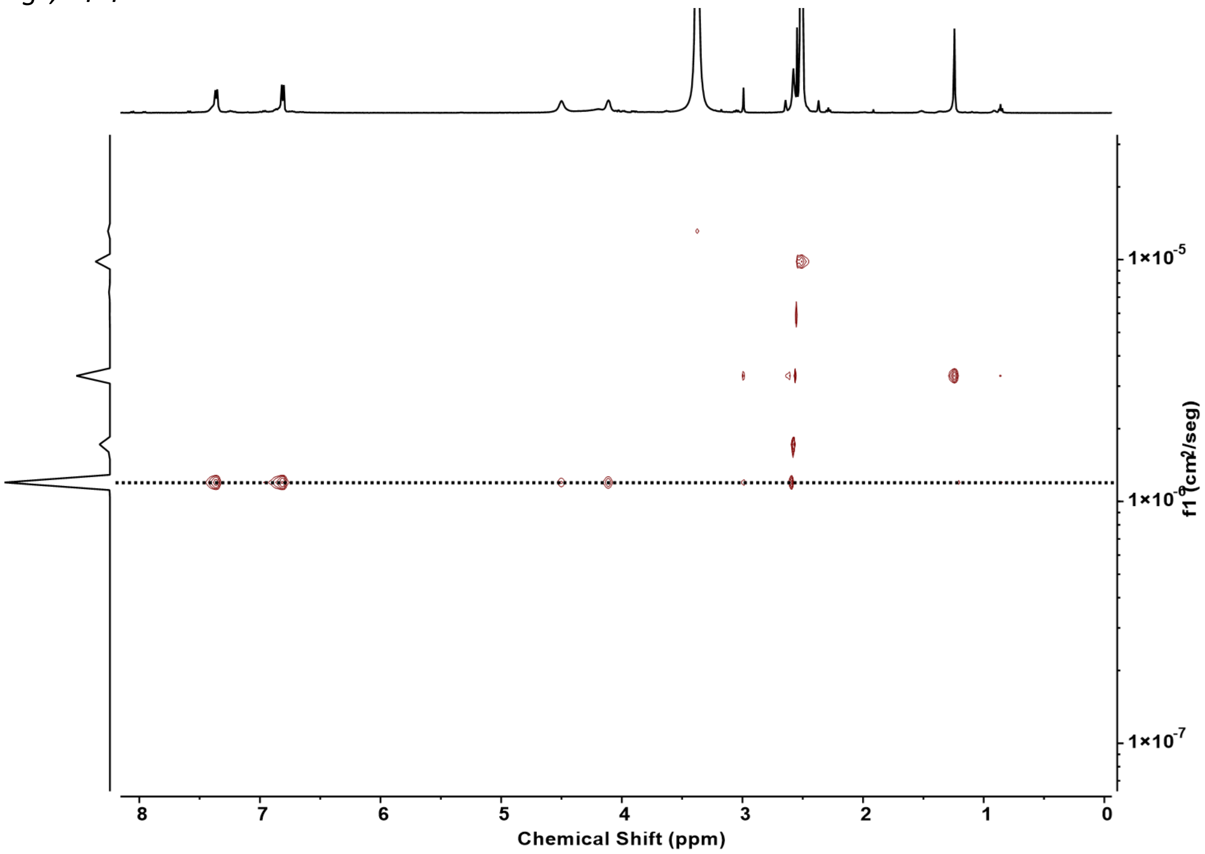

Figure S5. *^{1}H DOSY NMR spectrum of* ***A_4B_4*** *(500 MHz, DMSO, 298 K). The dashed line shows the diffusion of* ***p-A_4B_4****, with a diffusion coefficient of 1.18×10^{-10} m^2/s corresponding to a solvodynamic radius of 1.9 nm.*

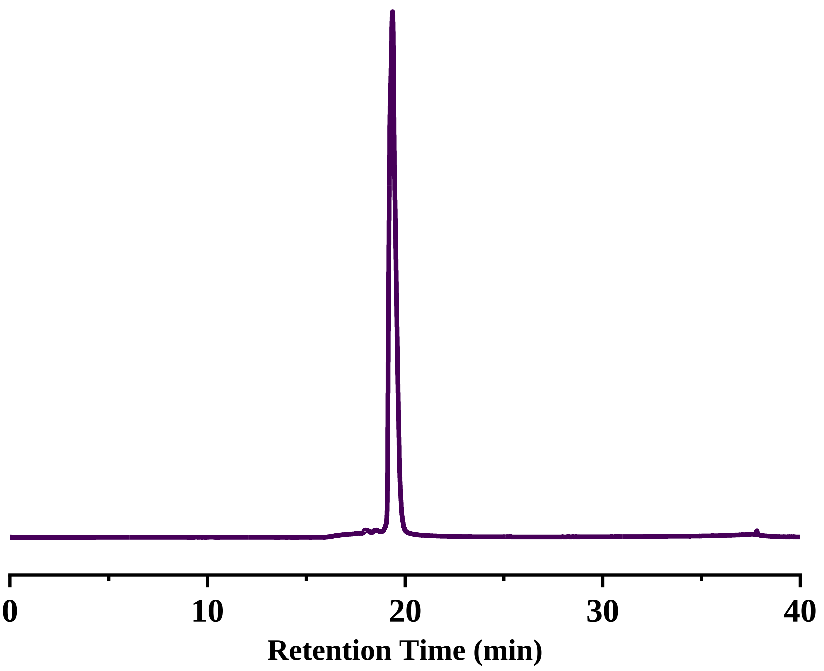

Figure S6. *Obtained pure HPLC chromatogram for* $\boldsymbol{A_4B_4}$*. Conditions: from 95% of water (0.1 % TFA) to 95% acetonitrile (0.1 % TFA) in 40 minutes.*

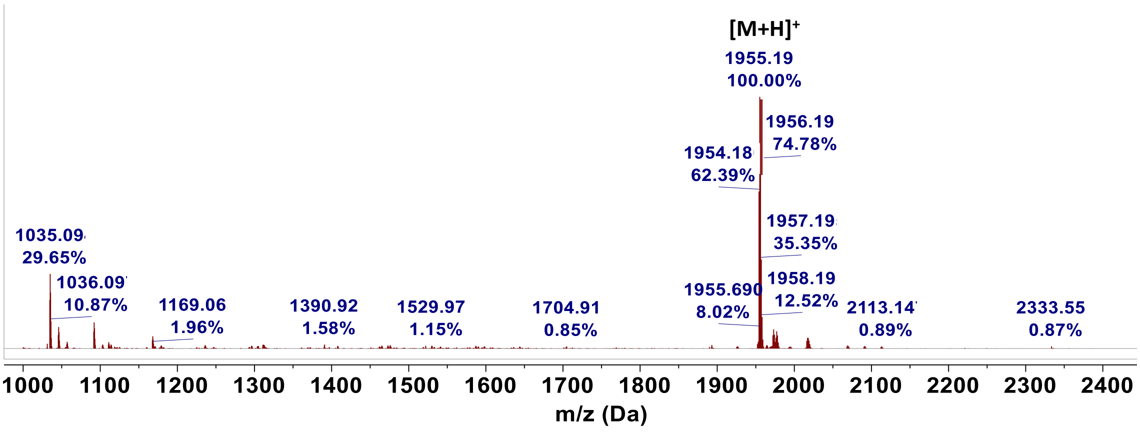

Figure S7. *Experimentally obtained HRMS for* $\boldsymbol{A_4B_4}$*.*

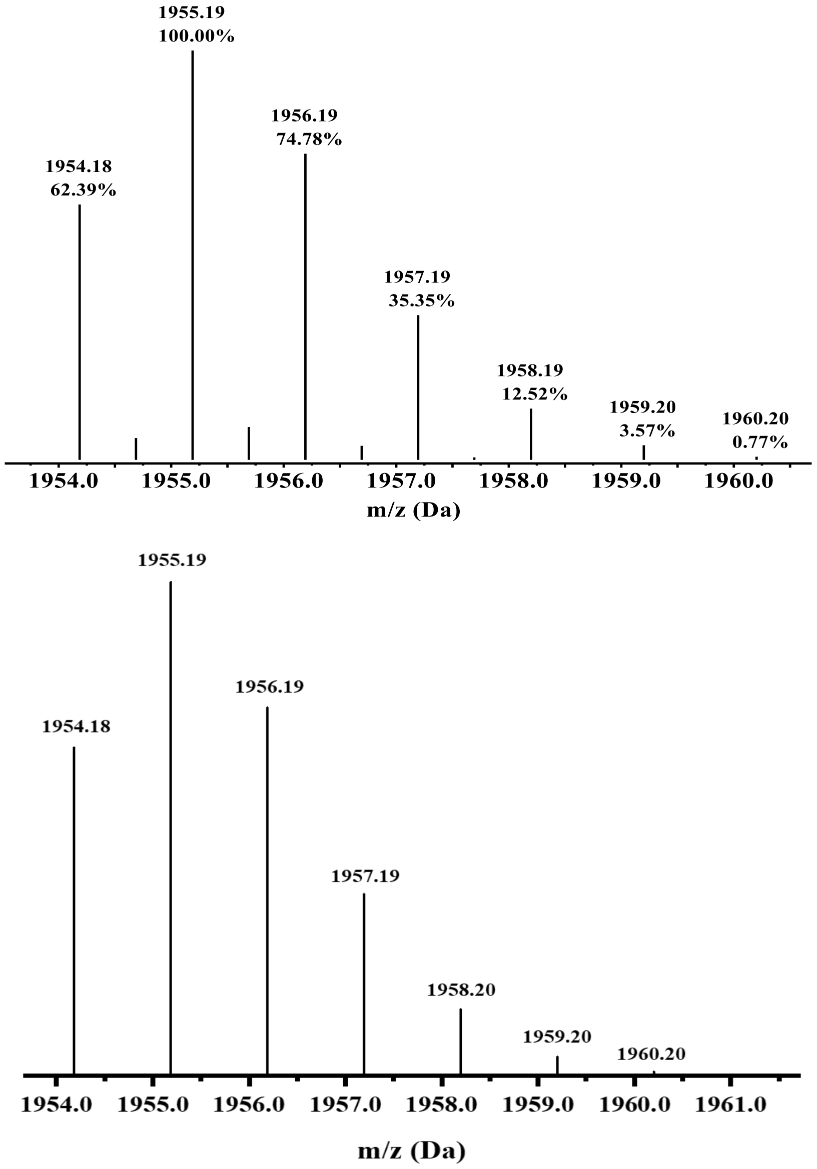

Figure S8. *Experimentally obtained (above) and predicted (below) HRMS for* ***A_4B_4****. Zoom in the $[M+H]^+$ peak.*

2.3. Functionalized molecular cage p-A_4B_4

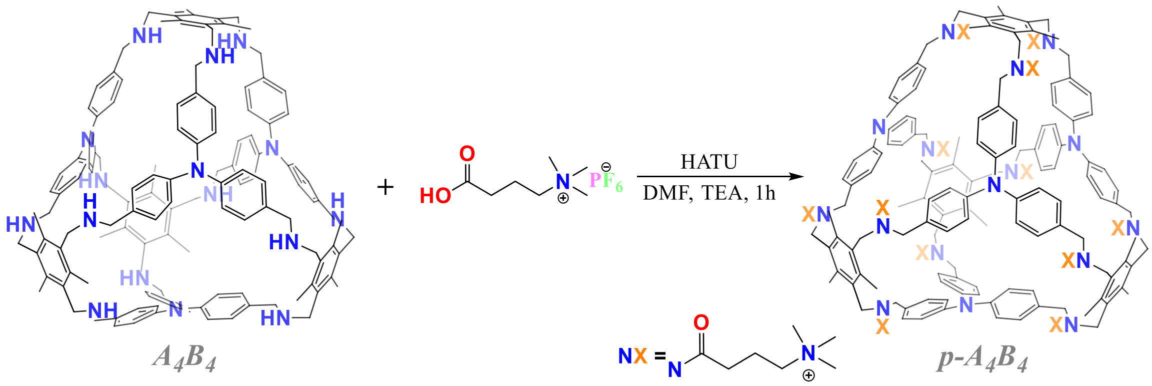

Scheme S4. *Synthetic procedure followed for the functionalization of **A_4B_4** to obtain **p-A_4B_4**, through amide bond formation.*

Previous to the modification of **A_4B_4**, the 3-carboxy-*N,N,N*-trimethylpropan-1-aminium chloride that was commercially available was treated with $NaPF_6$ in order to exchange the counterion because it was observed that this exchange does favor the reaction. Thus, 3-carboxy-*N,N,N*-trimethylpropan-1-aminium chloride was dissolved in water, where a solution of $NaPF_6$ was added, causing the precipitation of the salt, that was then dried.

3-carboxy-*N,N,N*-trimethylpropan-1-aminium hexafluorophosphate (75 mg, 15 eq.), HATU (146 mg, 15 eq.) and triethylamine (320 µL, 30 eq.) were dissolved in 5 mL of DMF and stirred at room temperature until the apparition of yellowish colour (around 5 min). Then, **A_4B_4** was added to the mixture (50 mg, 1 eq.). The resulting yellow solution was left stirring at room temperature for 1h. Purification was done by HPLC and 52 mg of product were obtained as a white solid (**p-A_4B_4**, 58 %).

HRMS(ESI+): *m/z* calc. for $C_{216}H_{311}N_{28}O_{12}$ $[M+7TFA]^{5+}$: 856.67. Found: 856.67.

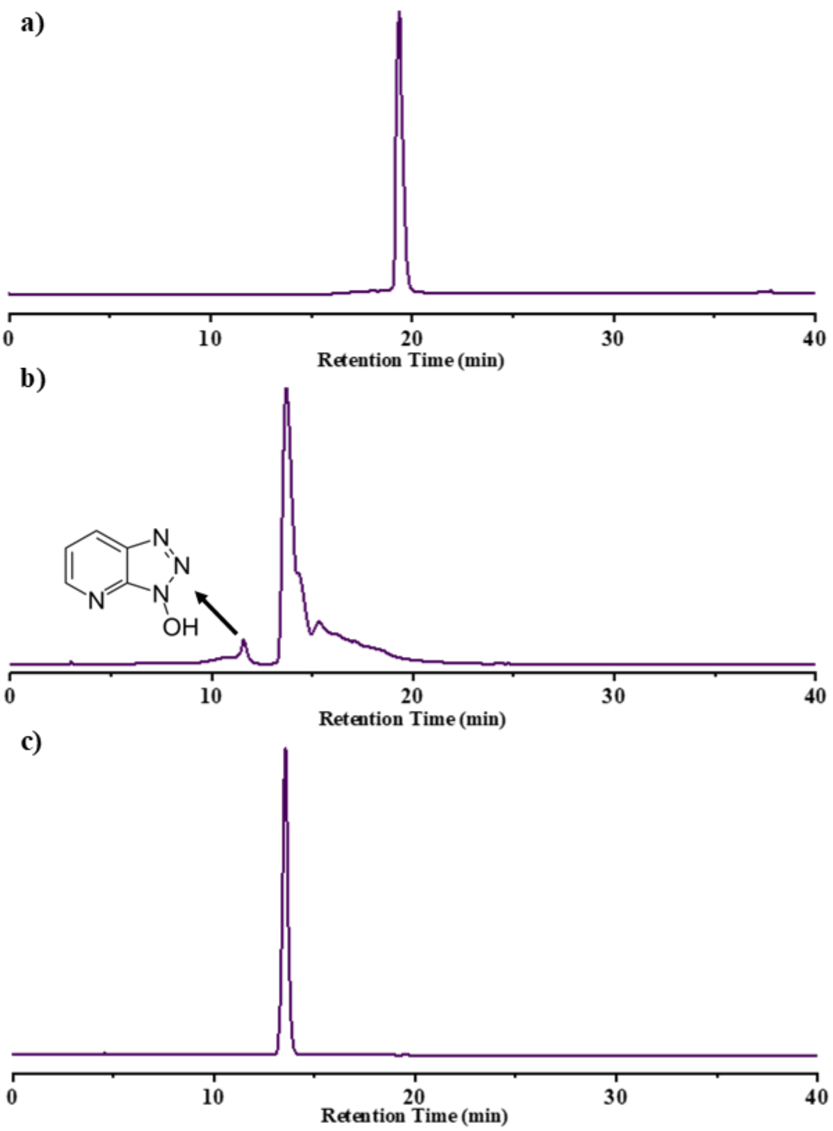

Figure S9. *Obtained HPLC chromatograms (absorbance at 300 nm) for a) pure* ***A₄B₄****, b) crude of the functionalization of* ***A₄B₄*** *to obtain* ***p-A₄B₄****. The peak at 12 minutes corresponds to HOAt and the formation of more polar reaction intermediates can be seen between 14 and 20 minutes, indicating an incomplete functionalization of the cage and c) purified* ***p-A₄B₄*** *product. Conditions: from 95% of water (0.1 % TFA) to 95% acetonitrile (0.1 % TFA) in 40 minutes.*

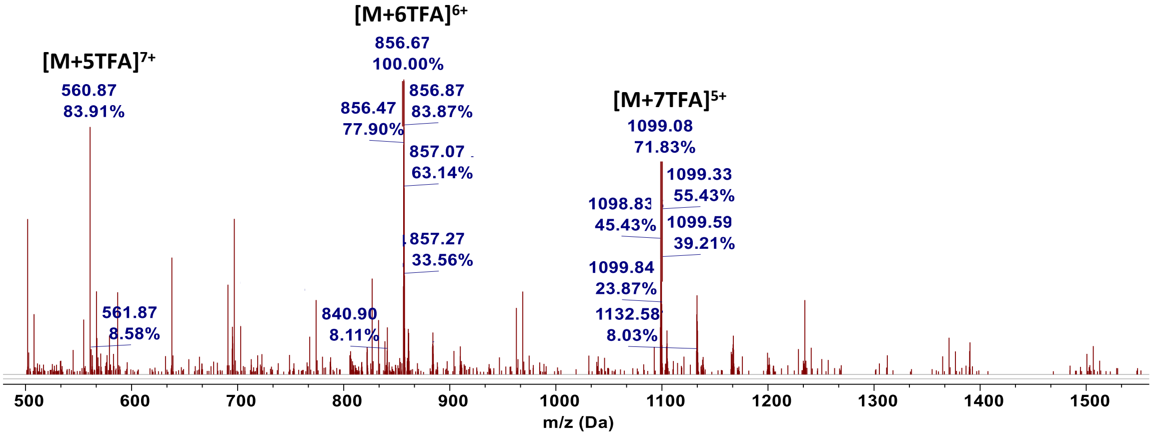

Figure S10. *Obtained HRMS for* ***p-A₄B₄*** *$[C_{216}H_{311}N_{28}O_{12}]^{12+}$. Peaks assigned on the spectrum.*

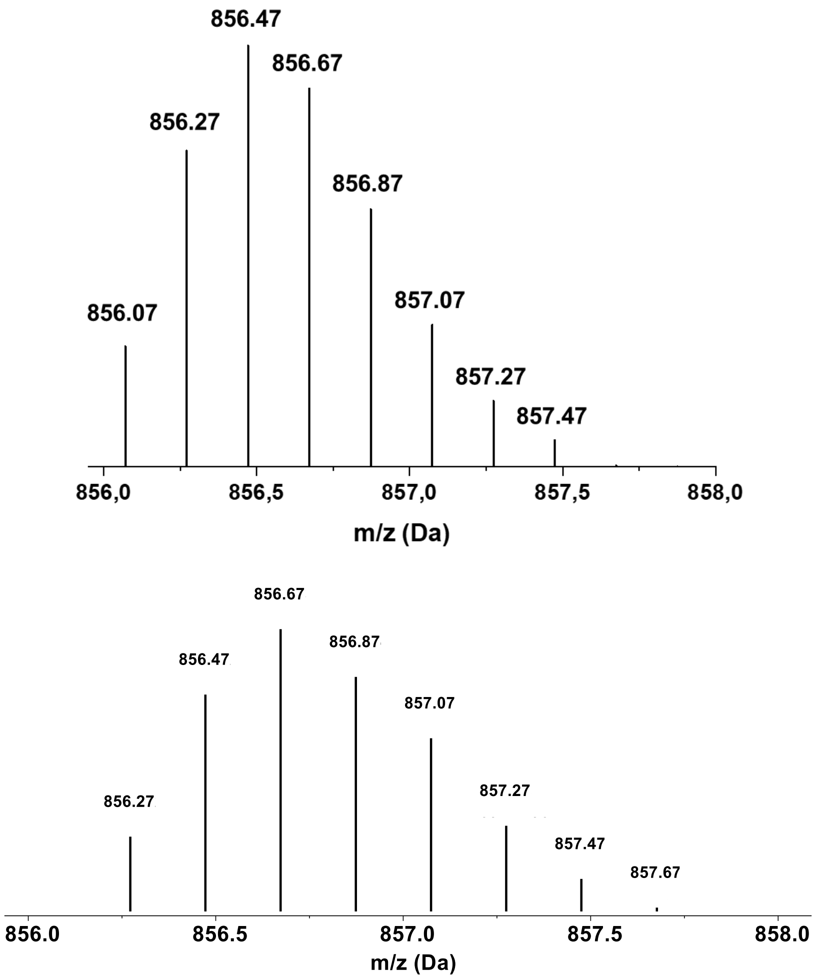

Figure S11. *Predicted (above) and experimentally obtained (below) HRMS for **p-A_4B_4**. Zoom in the $[M+6TFA]^{6+}$ peak.*

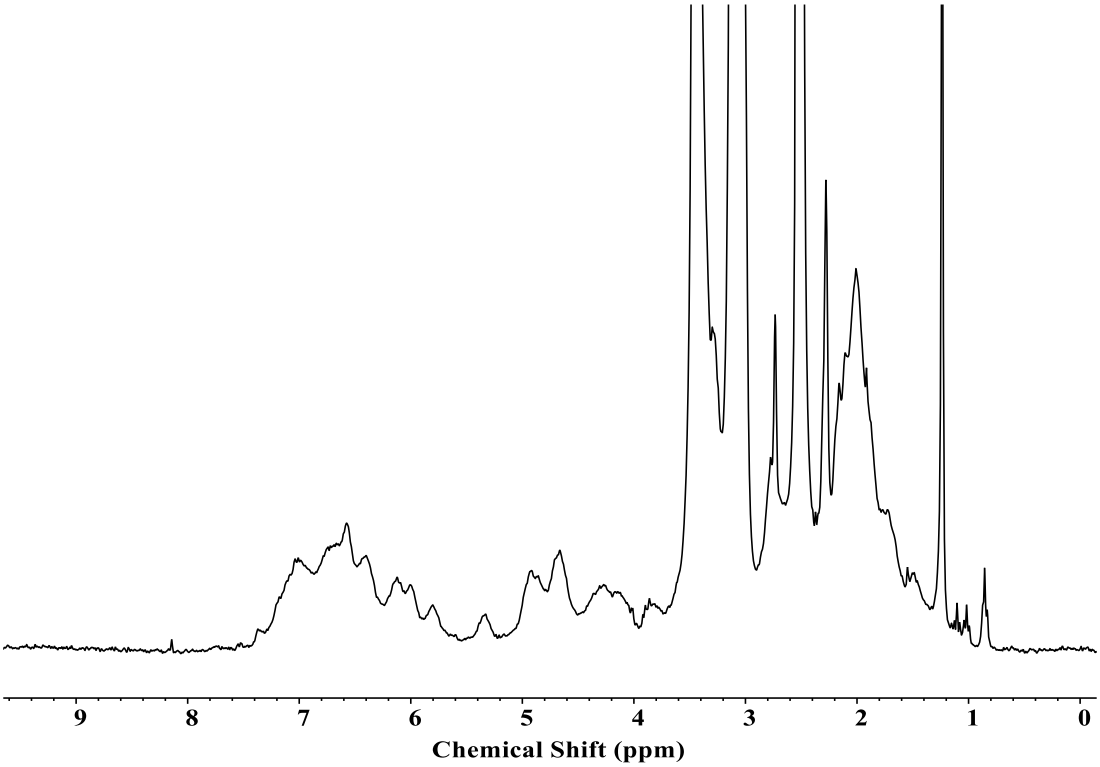

Figure S12. *^{1}H-NMR (500 MHz) characterization in DMSO of the positively charged molecular cage, **p-A_4B_4** (after purification) at room temperature.*

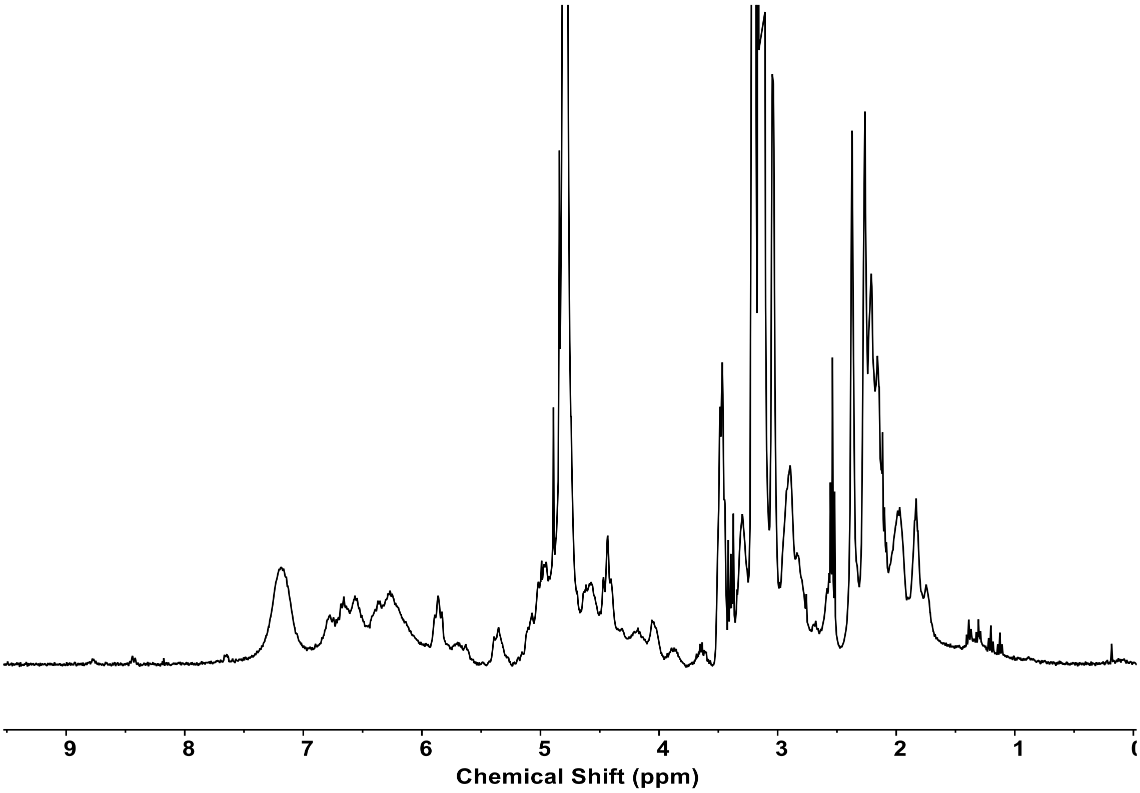

Figure S13. *^{1}H-NMR (500 MHz) characterization in D_2O of the positively charged molecular cage, **p-A_4B_4** (after purification), at room temperature.*

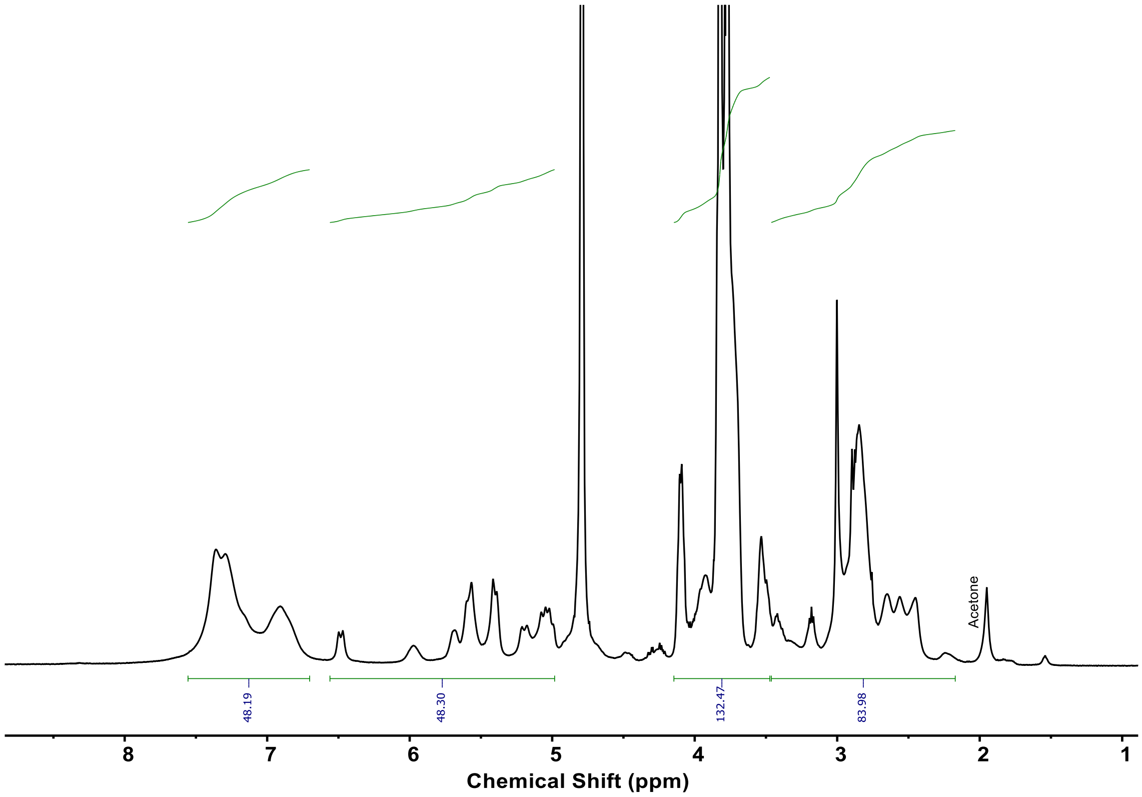

Figure S14. *^{1}H-NMR (500 MHz) characterization in D_2O of the positively charged molecular cage,* ***p-A_4B_4*** *(after purification),* at 90 °C.

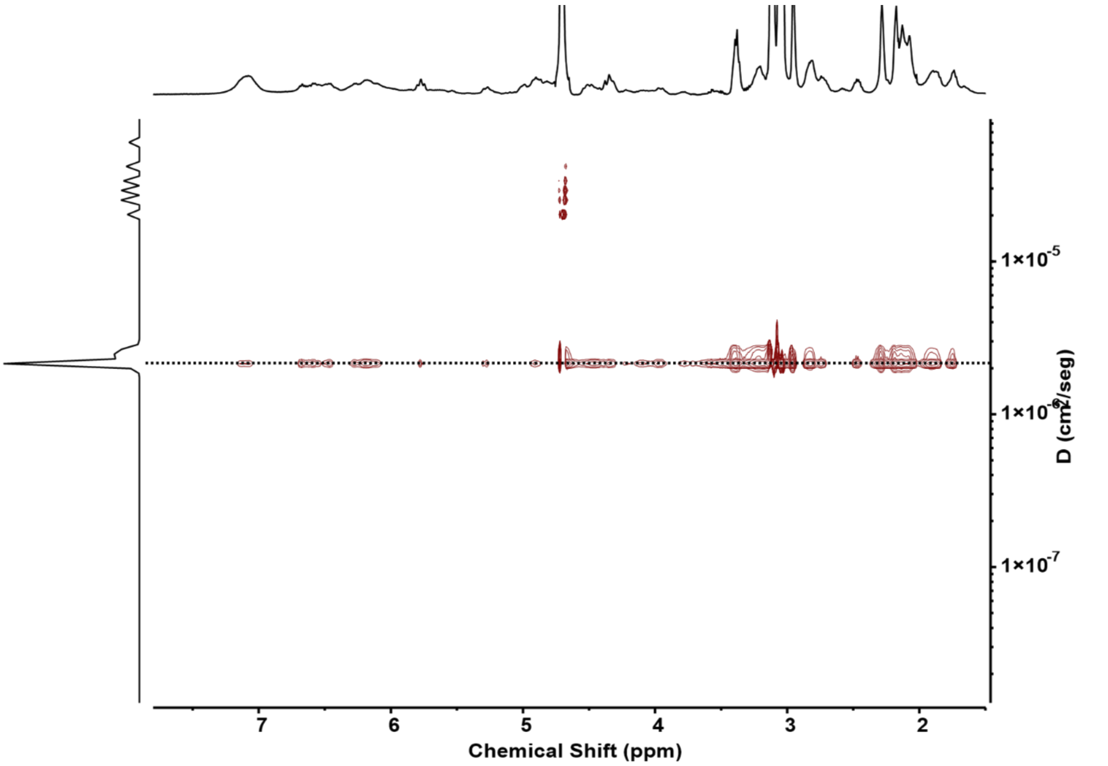

Figure S15. *^{1}H DOSY NMR spectrum of* ***p-A_4B_4*** *(500 MHz, D_2O, 298 K). The dashed line shows the diffusion of* ***p-A_4B_4****, with a diffusion coefficient of 2.15×10^{-10} m^2/s corresponding to a solvodynamic radius of 2.3 nm. Residual solvent signals for D_2O are shown at approximately 4.69 ppm.*

3. Interaction of p-A_4B_4 with hydrophobic anions

3.1. Tetra(4-sulfonatophenyl)porphyrin

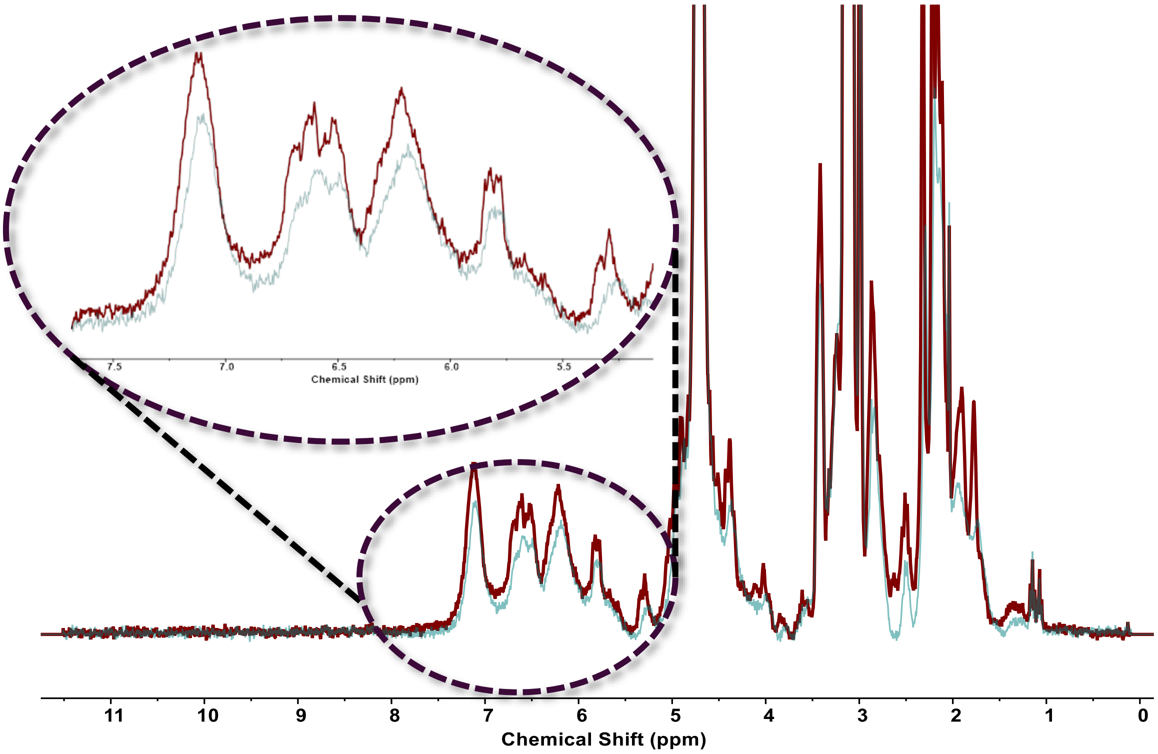

Figure S16. *^{1}H-NMR (300 MHz, 298 K) of* ***p-A_4B_4*** *(red) superimposed with the obtained spectrum after the addition of 1 equivalent of tetra(4-sulfonatophenyl)porphyrin (blue). Zoom in the aromatic region from* ***p-A_4B_4,*** *showing interaction (displacement of the signals) but not precipitation. [tetra(4-sulfonatophenyl)porphyrin] = 0.5 mM*

3.2. 8-hydroxypyrene-1,3,6-trisulfonate

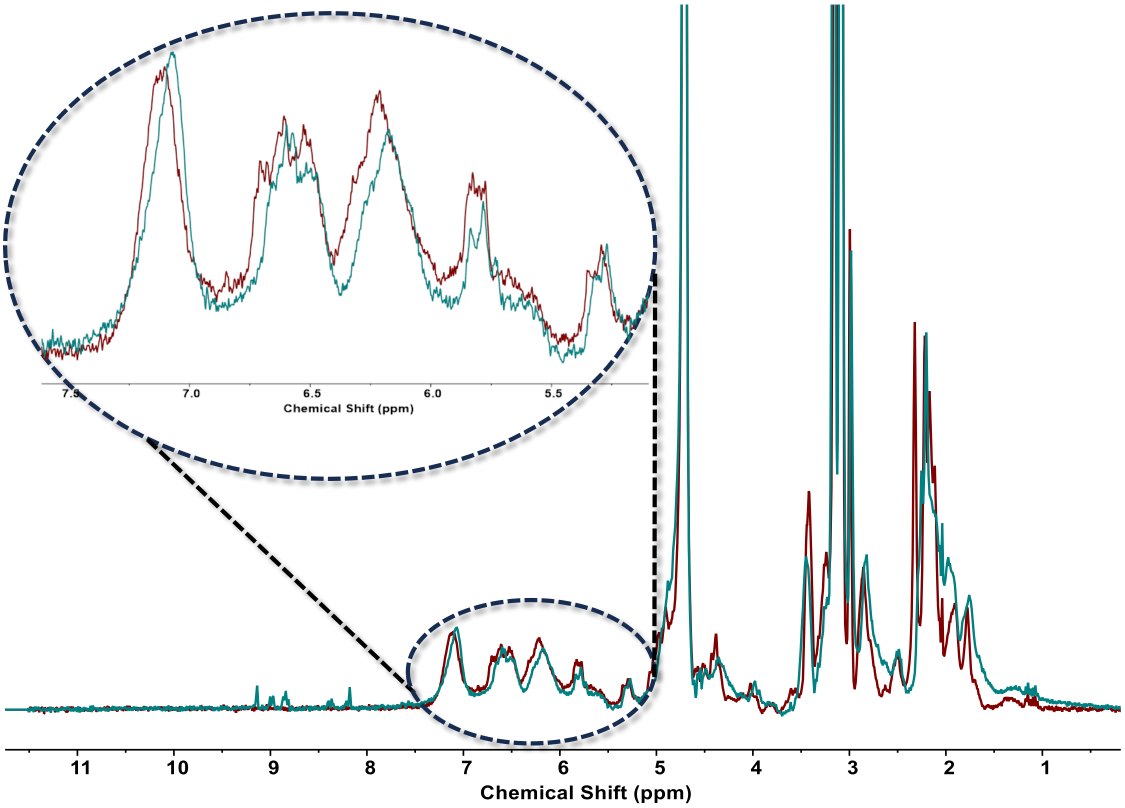

Figure S17. *^{1}H-NMR (300 MHz, 298 K) of* ***p-A$_4$B$_4$*** (red) *superimposed with the obtained spectrum after the addition of 1 equivalent of 8-hydroxypyrene-1,3,6-trisulfonate* (blue). *Zoom in the aromatic region from* ***p-A$_4$B$_4$****, showing interaction (displacement of the signals) but not precipitation. [8-hydroxypyrene-1,3,6-trisulfonate] = 0.5 mM*

3.3. p-toluenesulfonate

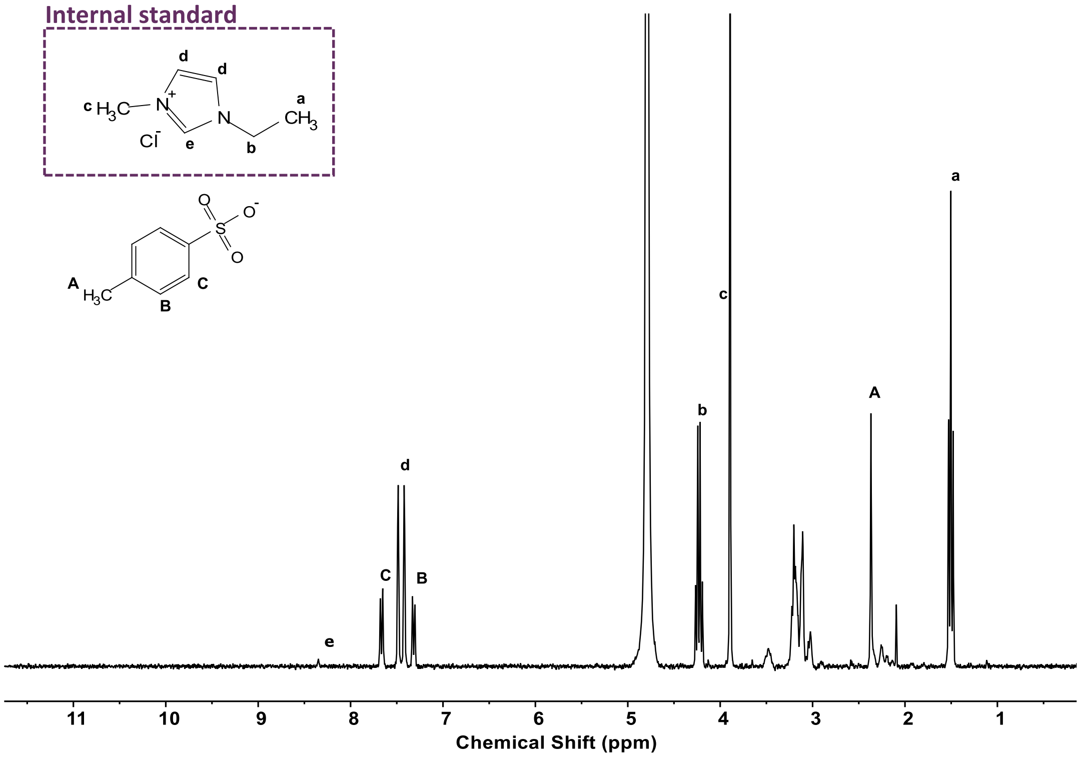

Figure 18. *^{1}H-NMR (300 MHz, 298 K) of p-toluene sulfonate (1 mM) with the reference (3 mM), in D_2O.*

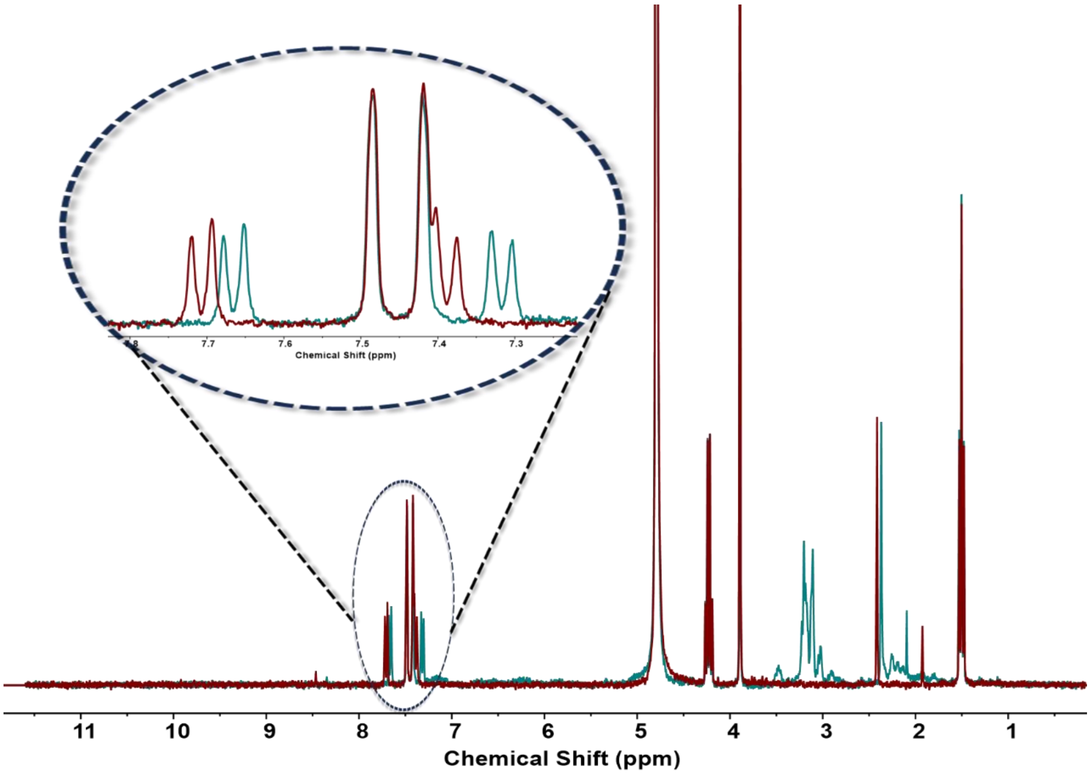

Figure S19. *^{1}H-NMR (300 MHz, 298 K) of p-toluene sulfonate (1 mM) with the reference (3 mM) superimposed with the obtained spectrum after the addition of 0.5 equivalents*

*of **p-A₄B₄** (blue). Zoom in the aromatic region from the p-toluenesulfonate, showing interaction (displacement of the signals) but not precipitation between it and **p-A₄B₄.***

4. Interaction of p-A_4B_4 with surfactants

The general protocol used to titrate surfactants with the positively charged cage is described as follows: A solution of 1 mM surfactant and 3 mM of internal standard (1-ethyl-3-methylimidazolium chloride) was titrated by adding increasing amounts of **p-A_4B_4**, from 0 to 0.05 equivalents (unless stated otherwise). The progress of the titration was followed by ^{1}H-NMR, where the terminal methyl group of each of the surfactants was monitored. Note that all the experiments were carried using 1-Ethyl-3-methyl-1H-imidazol-3-ium chloride as the reference compound.

4.1. 6C surfactant

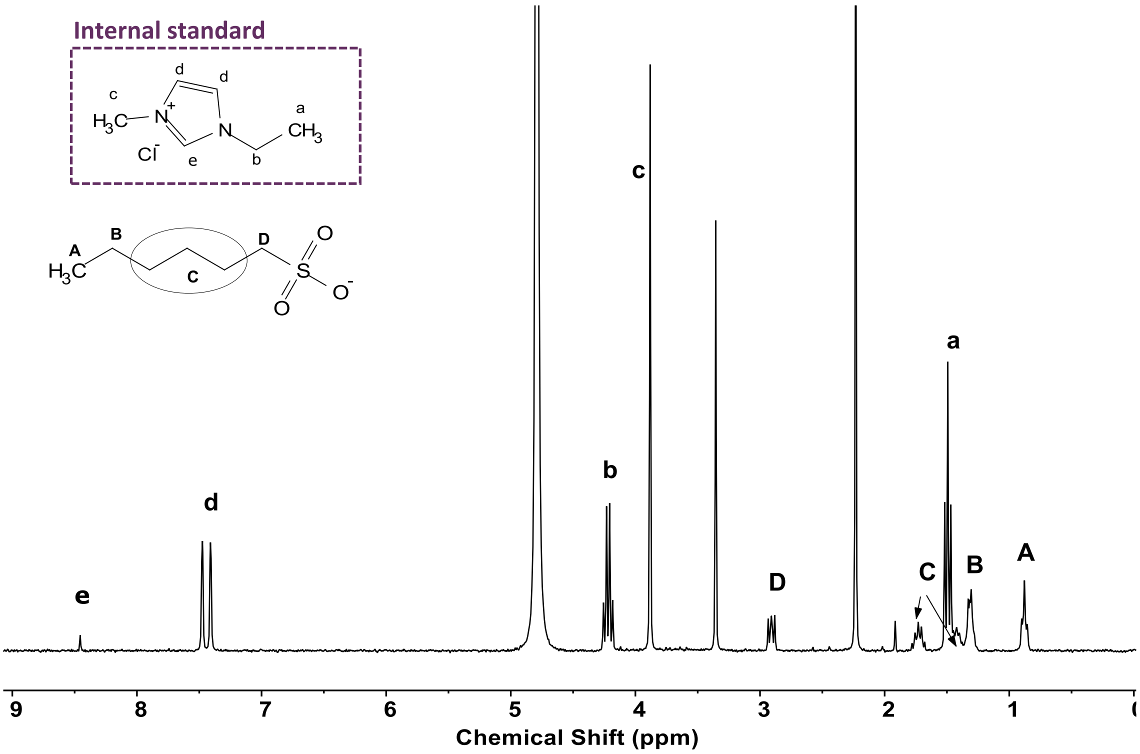

Figure 20. *^{1}H-NMR (300 MHz, 298 K) of 1 mM 6C surfactant and 3 mM reference, in D2O.*

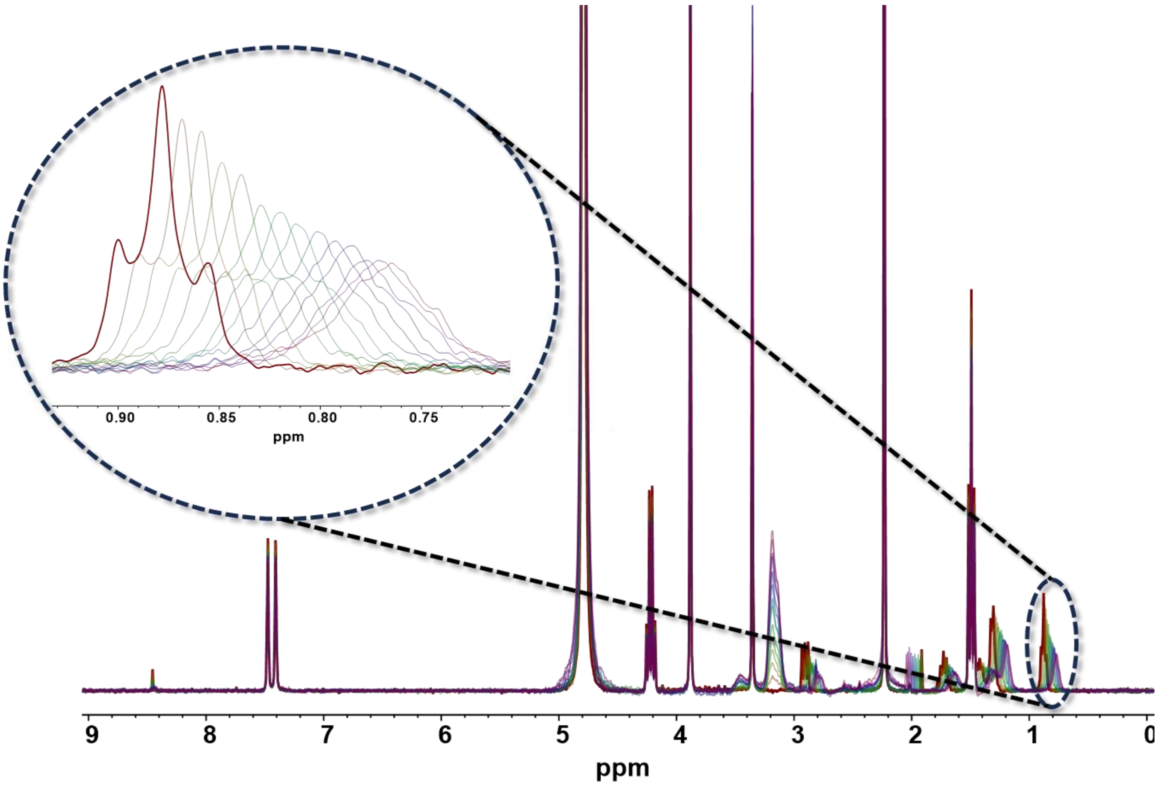

Figure S21. *^{1}H-NMR (300 MHz, 298 K) of 1 mM* ***6C*** *surfactant and 3 mM reference superimposed with the obtained spectrum after the addition of increasing amounts of* ***p-A$_4$B$_4$****, from 0.01 to 0.3 equivalents. Zoom in the surfactant's terminal methyl group signals showing interaction (displacement of the signals) but no precipitation.*

4.2. SDS surfactant

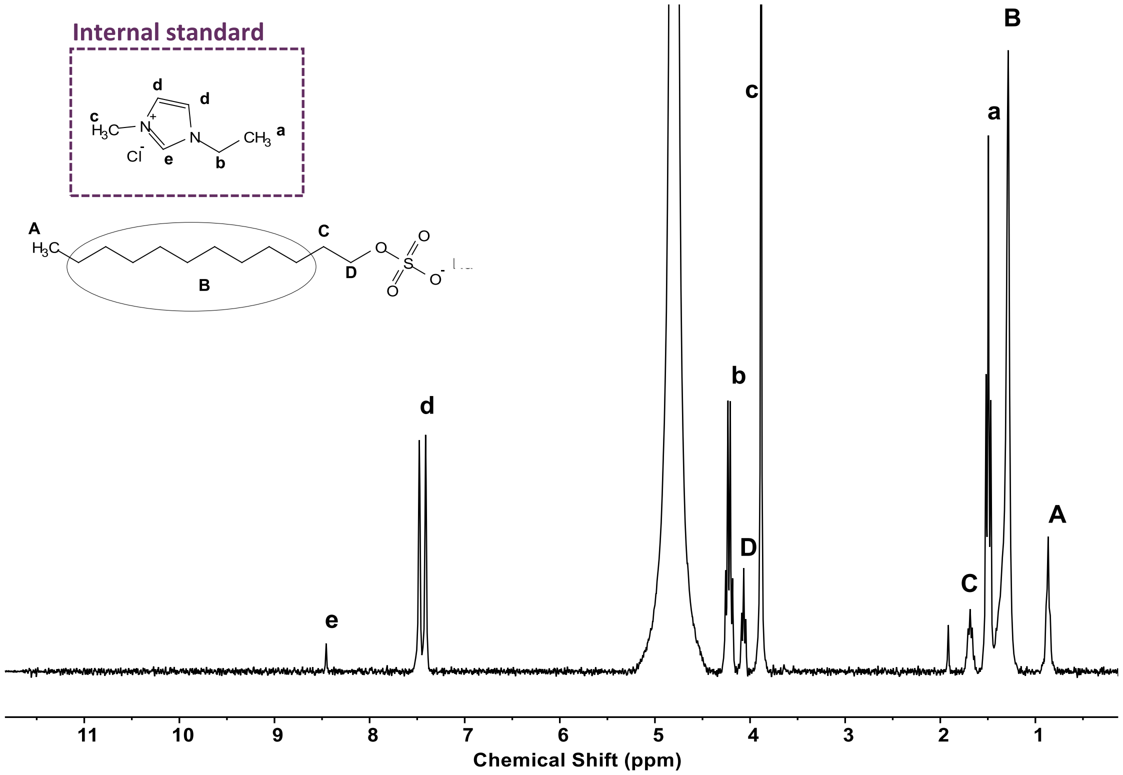

Figure 22. *^{1}H-NMR (300 MHz, 298 K) of 1 mM SDS and 3 mM reference in D$_2$O.*

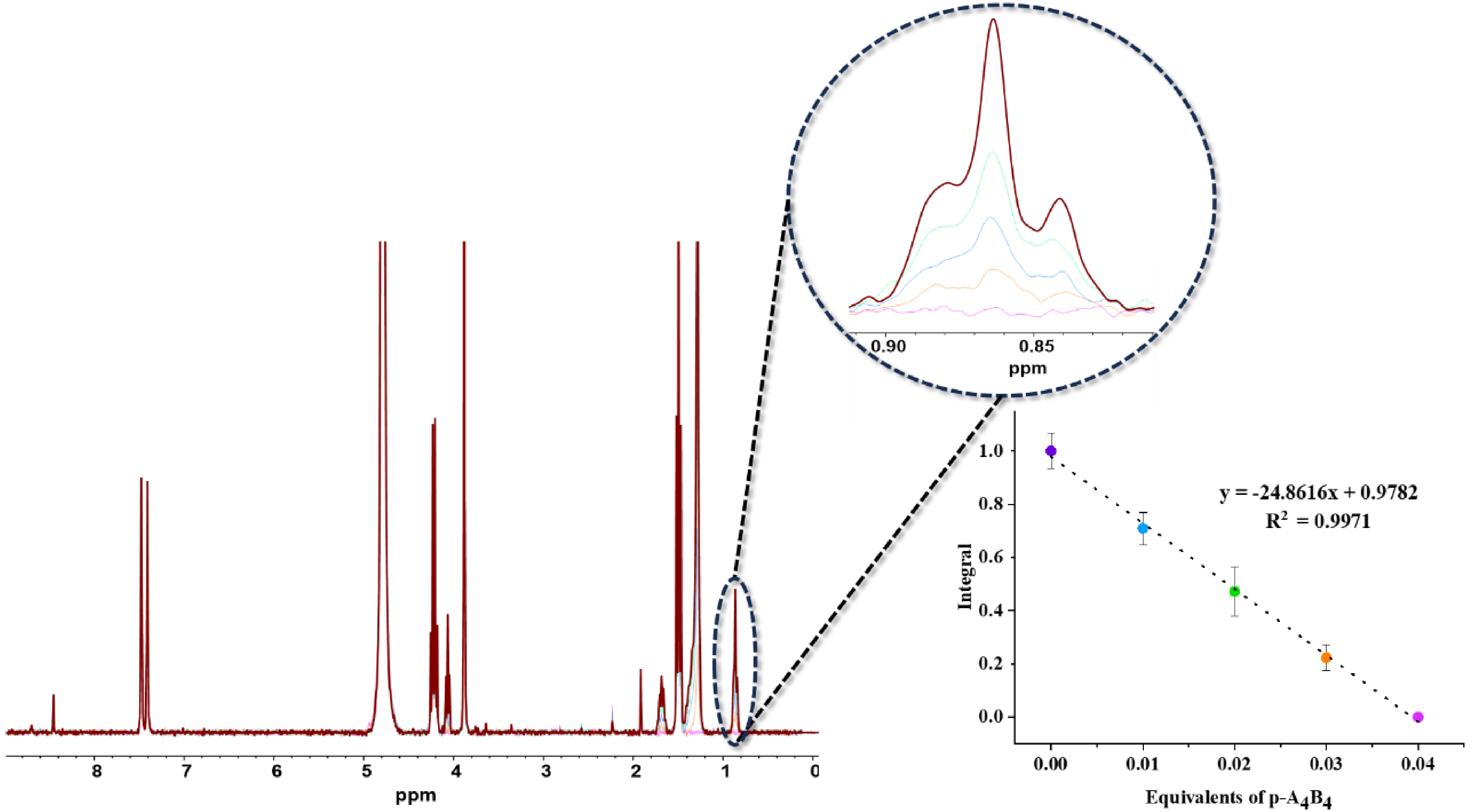

Figure S23. *^{1}H-NMR (300 MHz, 298 K) of 1 mM SDS and 3 mM reference superimposed with the obtained spectrum after the addition of up to 0.04 equivalents of* ***p-A₄B₄****. Zoom in the surfactant's terminal methyl group signals, showing signal decay until the complete disappearance of the surfactant in solution. The graph represents the decrease of the peak integral upon addition of* ***p-A₄B₄.***

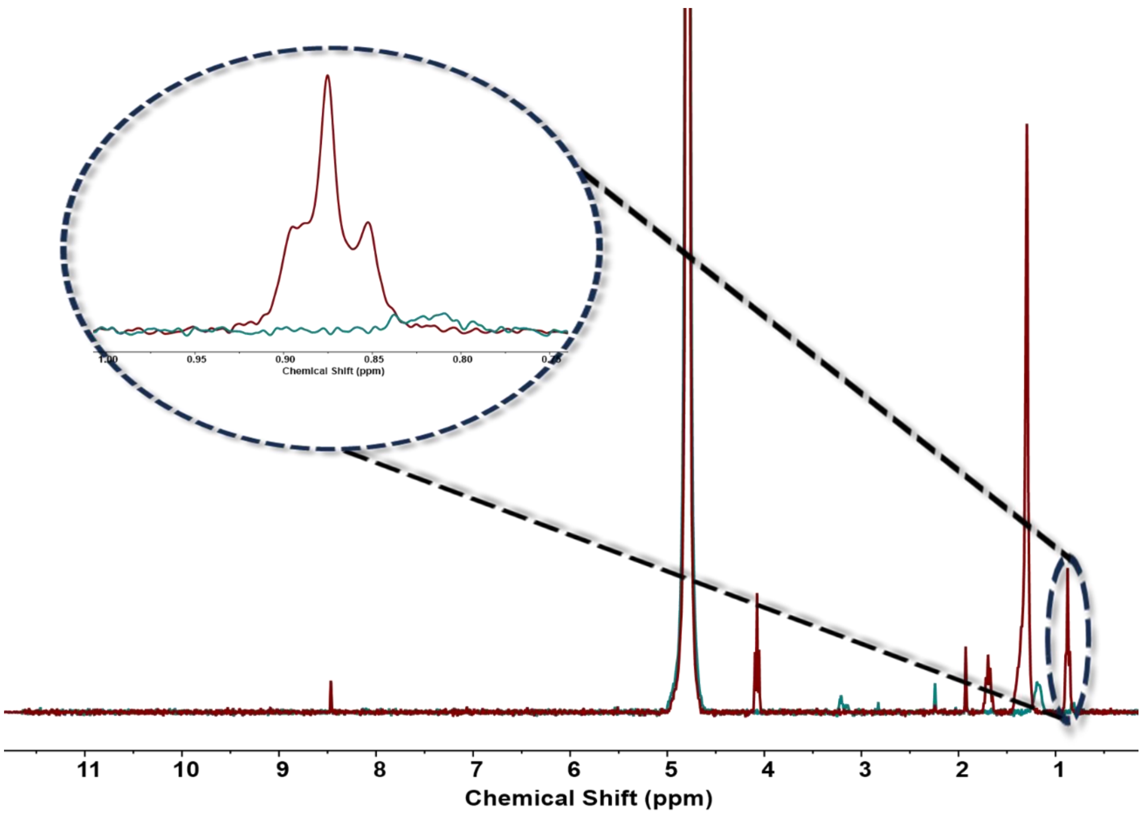

Figure S24. *^{1}H-NMR (300 MHz, 298 K) of 1 mM SDS in 10 mM PBS buffer superimposed with the obtained spectrum after the addition of 0.04 equivalents of* ***p-A₄B₄****, proving that there is precipitation in complex media.*

4.3. 16C surfactant

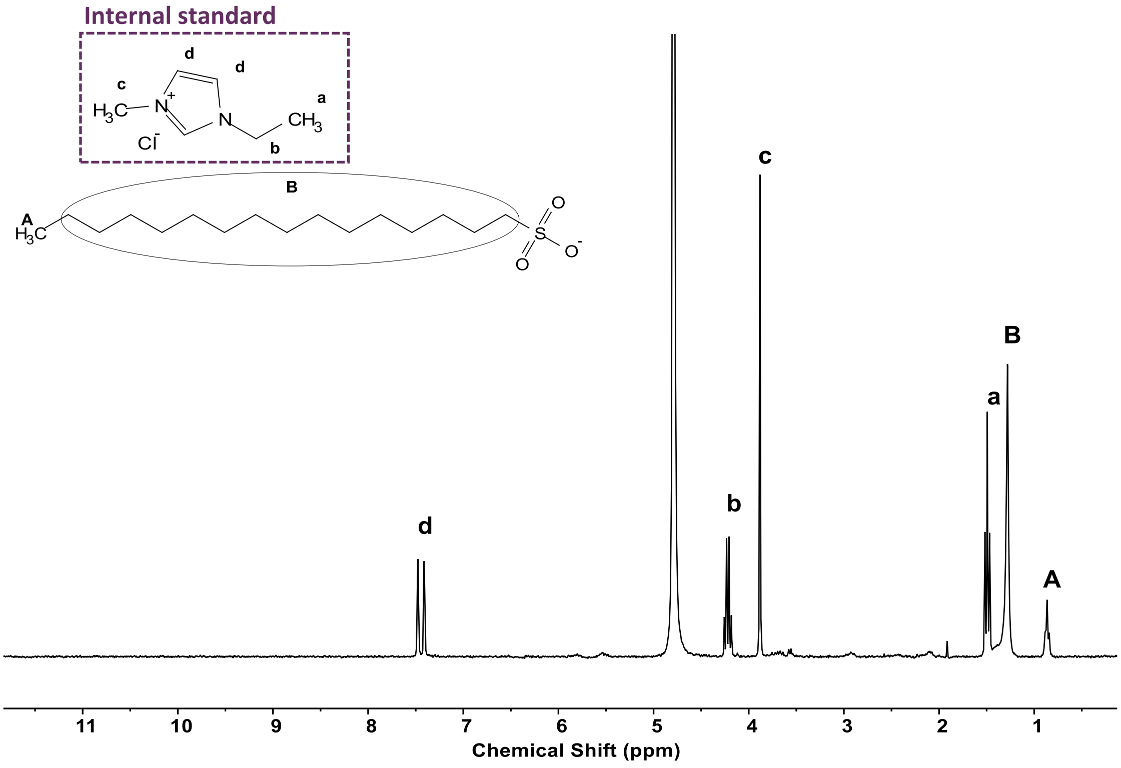

Figure 25. *^{1}H-NMR (300 MHz, 298 K) of 1 mM* ***16C*** *and 3 mM reference, in D_2O.*

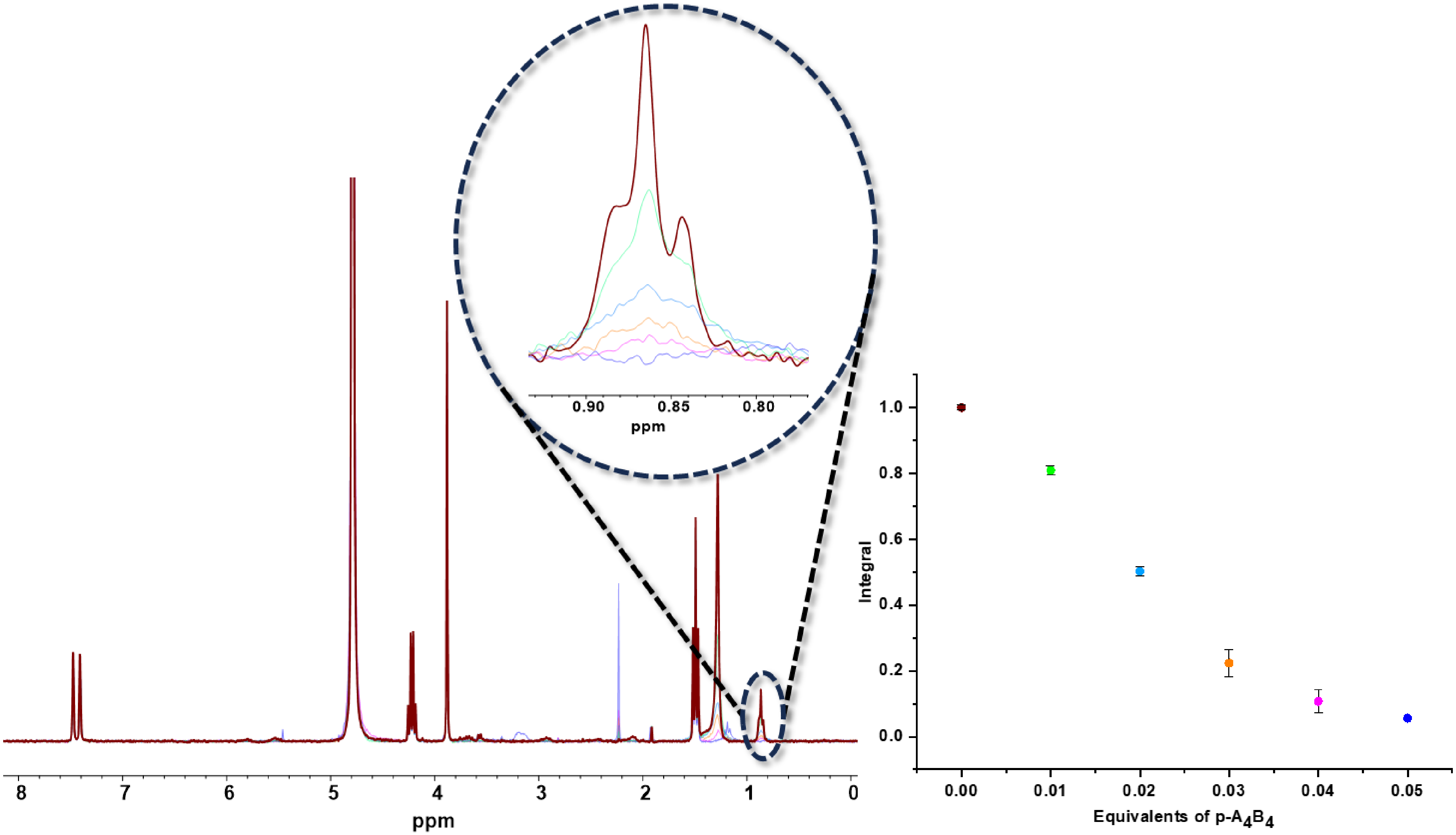

***Figure S26**. ^{1}H-NMR (300 MHz, 298 K) of 1 mM **16C** and 3 mM reference superimposed with the obtained spectrum after the addition of up to 0.05 equivalents of **p-A_4B_4**. Zoom in the surfactant's terminal methyl group signals, showing signal decay until the complete disappearance of the surfactant in solution. The graph represents the decrease of the peak integral upon addition of **p-A_4B_4**.*

4.4. Sodium oleate

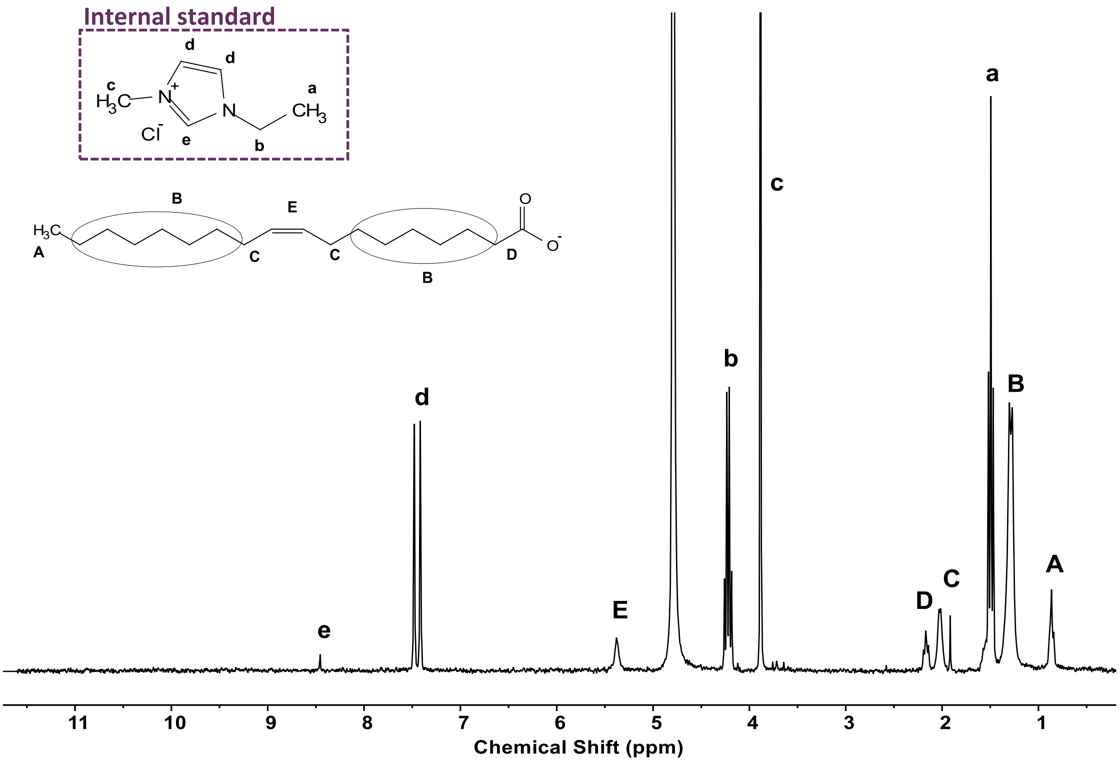

Figure 27. *^{1}H-NMR (300 MHz, 298 K) of 1 mM sodium oleate and 3 mM reference, in D_2O.*

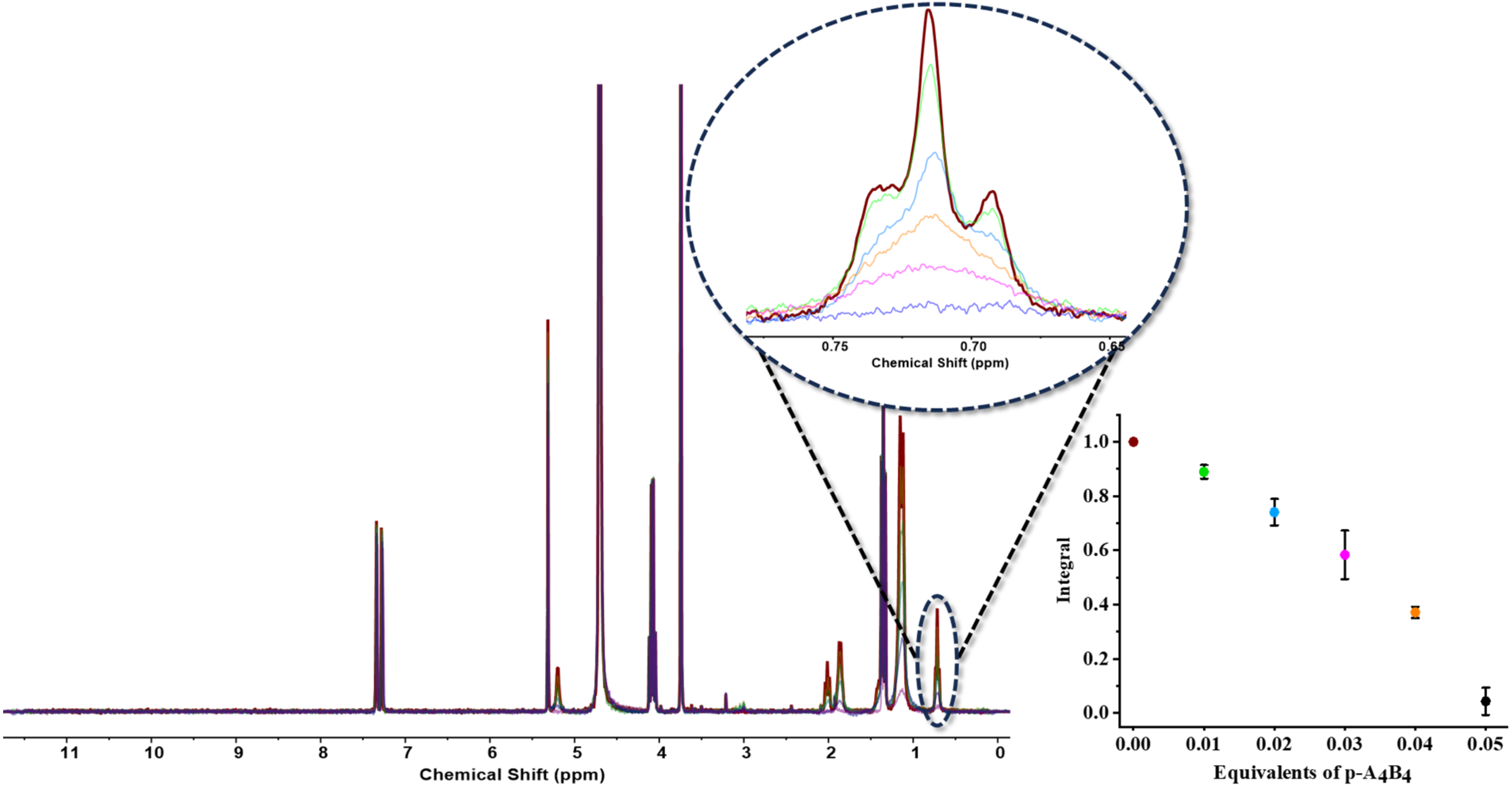

Figure S28. *^{1}H-NMR (300 MHz, 298 K) of 1 mM sodium oleate and 3 mM reference and superimposed with the obtained spectrum after the addition of up to 0.05 equivalents of* ***p-A$_4$B$_4$****. Zoom in the surfactant's terminal methyl group signals, showing signal decay until the complete disappearance of the surfactant in solution. The graph represents the decrease of the peak integral upon addition of* ***p-A$_4$B$_4$****.*

4.5. CTAB

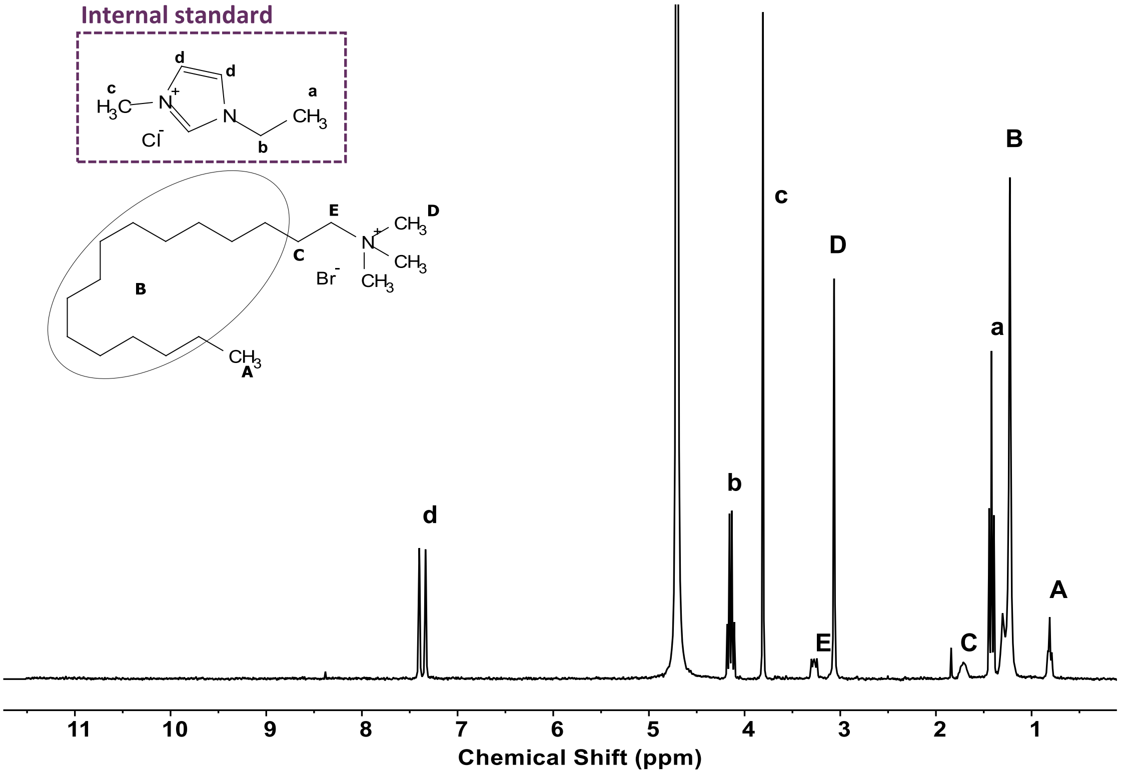

Figure 29. *^{1}H-NMR (300 MHz, 298 K) of 1 mM CTAB and 3 mM reference, in D_2O.*

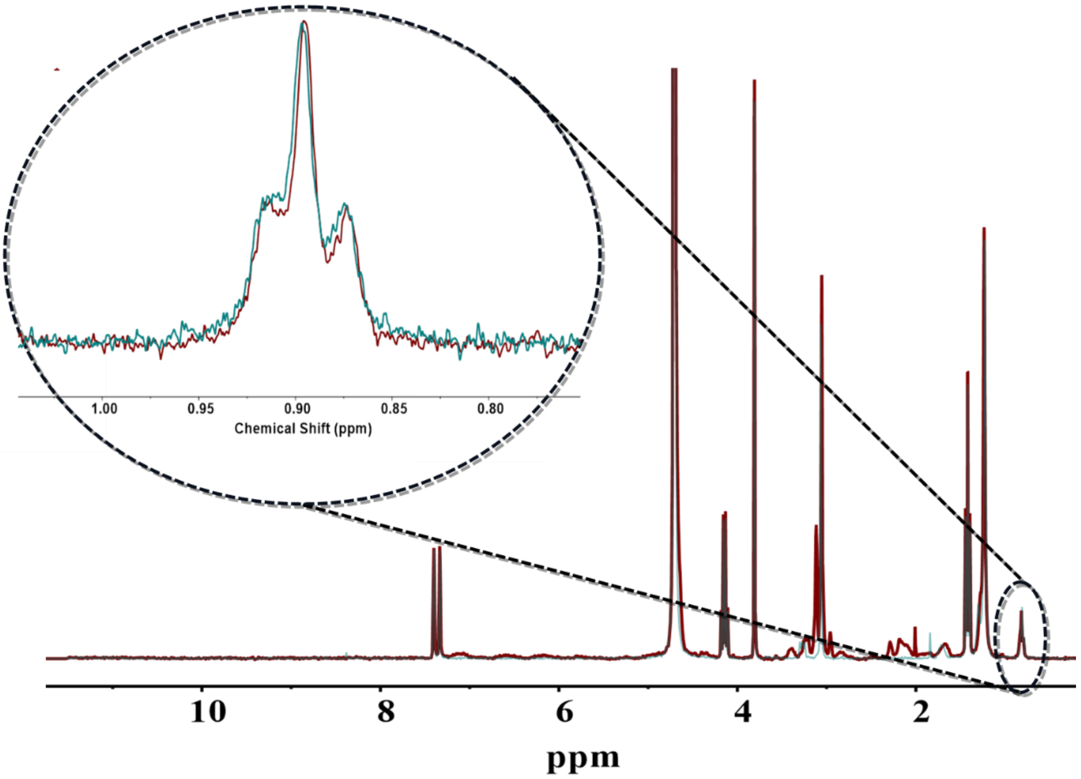

Figure S30. *^{1}H-NMR (300 MHz, 298 K) of 1 mM CTAB and 3 mM reference and superimposed with the obtained spectrum after the addition of 0.05 equivalents of **p-A_4B_4** (blue). Zoom in the surfactant's terminal methyl group signals, not showing interaction or precipitation.*

4.6. n-Dodecyl-β-D-maltoside

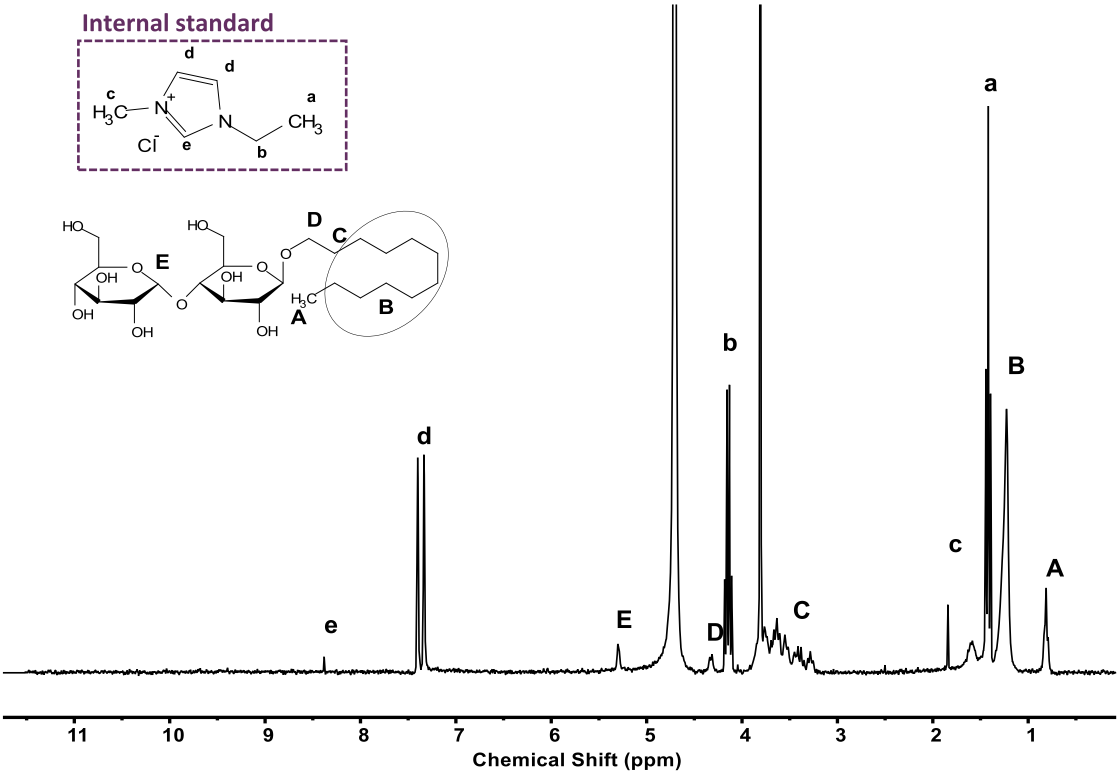

Figure 31. *1H-NMR (300 MHz, 298 K) of 1 mM n-Dodecyl-β-D-maltoside surfactant and 3 mM reference, in D_2O.*

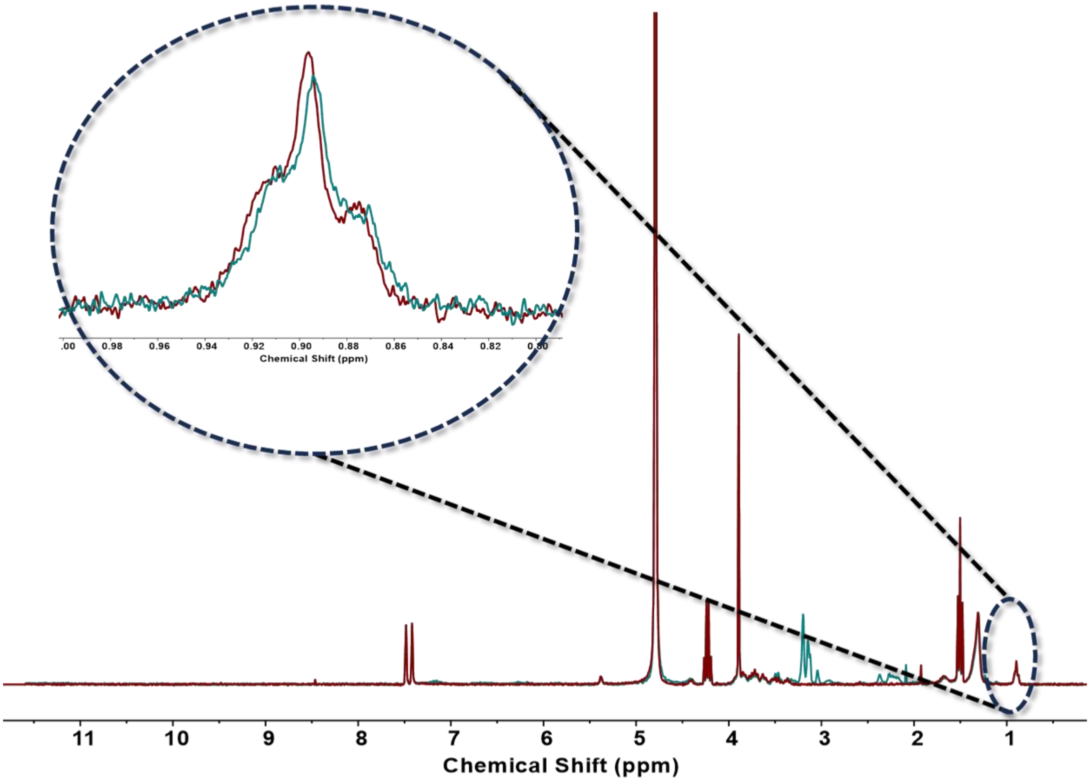

Figure S32. *^{1}H-NMR (300 MHz, 298 K) of 1 mM n-Dodecyl-β-D-maltoside surfactant and 3 mM reference and superimposed with the obtained spectrum after the addition of 0.05 equivalents of* ***p-A_4B_4*** *(blue). Zoom in the surfactant's terminal methyl group signals, not showing interaction nor precipitation.*

5. Computational methods: MD simulations

All MD simulations were conducted using the LAMMPS simulation package (stable release 23 Jun 2022) (2). Force field parameters for the inner region of **p-A_4B_4** were taken from the all-atom Optimized Potentials for Liquid Simulations (OPLS-AA) force field (3,4), while parameters for the outer and cationic regions of **p-A_4B_4** were taken from the Canongia Lopes & Padua (CL&P) force field (5,6). Parameters for one atom type, an aromatic carbon bonded to a tertiary amine, were not present in either force field. This atom type was assigned a partial charge to maintain a +12 formal charge on **p-A_4B_4**, and OPLS-AA parameters for a standard aromatic carbon were used for all other interactions. Force field parameters for the neutral portions of surfactants were taken from the OPLS-AA force field, while parameters for the charged head groups were taken from the CL&P force field. All parameters for counterions were obtained from the CL&P force field. Water was modeled using the rigid TIP4P model (7). Real-space non-bonded interactions were truncated at 12.0 Å. Long-range electrostatics were handled using the particle-particle-particle-mesh Ewald summation method with a convergence accuracy of 10^{-5} (8).

Cubic simulation cells were used for all simulations with periodic boundary conditions applied in every dimension. Simulations containing a single **p-A_4B_4** molecule used a box length of 90 Å, while simulations containing three **p-A_4B_4** molecules used a box length of 130 Å. Simulation cells were initially prepared by inserting the desired molecules with their appropriate counterions. Sodium cations were used as the counterions for anionic surfactants, bromine anions were used as the counterions for CTAB, and 12 trifluoroacetic acid molecules were used as counterions for **p-A_4B_4**. All systems were solvated with randomly inserted water molecules to achieve a density of 1000 kg/m^3. Water molecules were not included if they could not be placed a distance of at least 1.3 Å from other molecules in the system. All systems underwent 10,000 steps of energy minimization. Systems were initially relaxed for 50 ps at 300 K using a Nosé-Hoover thermostat (9) with a damping constant of 100 fs. The systems were then subject to a brief equilibration for 100 ps at 300 K and 1 bar using a Nosé-Hoover thermostat and barostat. Given that rigid TIP4P water required separate treatment of dynamics, *NPT* equilibration coupled only water molecules to the Nosé-Hoover barostat, while all

remaining molecules were coupled to a Nosé-Hoover thermostat. All production run simulations used the same conditions as the *NPT* equilibration. Equations of motion were evolved using a velocity-Verlet integration scheme with a 1 fs timestep.

For simulations involving one **p-A_4B_4** and 48 surfactant molecules, three replicates of the system were simulated for 80 ns with different initial velocities. The number of attached surfactants was calculated every 10 ps. For the binding analysis in Figure 4a, attached surfactants were defined as those surfactants in the same cluster as the **p-A_4B_4** molecule. All clusters were defined using a 3 Å cutoff. For the analysis in Figure 4d, only those surfactant molecules within 3 Å of the **p-A_4B_4** molecule were considered.

For simulations involving one **p-A_4B_4** molecule and initially one surfactant molecule, three replicates of the system were simulated using different initial velocities. Surfactants and counterions were gradually inserted into the simulation box by placing a surfactant and counterion into the simulation, deleting any water molecules within a 1.5 Å cutoff, minimizing the system for 5 ps, and then simulating for 2 ns. This cycle was repeated 24 times for a total duration of 48 ns. Attached surfactants were defined as those surfactants in the same cluster as the **p-A_4B_4** molecule.

For simulations involving three **p-A_4B_4** molecules, there were 83 surfactant molecules and the requisite number of counterions. These molecules were initialized so that they all belonged to the same cluster before relaxation and brief equilibration. Following equilibration, three replicates of the system were simulated for 30 ns with different initial velocities. This approach was taken to overcome the expected long timescales associated with **p-A_4B_4** diffusion and should represent a bound on the true behavior of the physical system.

6. References

[1] R. L. Greenaway, et al., *Nat. Commun*. **2018**, *9*, 2849.

[2] A. P. Thompson, et al., *Comput. Phys. Commun.* **2022**, *271*, 108171.

[3] W. L. Jorgensen, D. S. Maxwell, J. Tirado-Rives, *J. Am. Chem. Soc.* **1996**, *118*, 11225.

[4] S. W. Siu, K. Pluhackova, R. A. Bockmann, J*. Chem. Theory Comput.* **2012**, *8*, 1459.

[5] J. N. Canongia Lopes, A. A. H. Pádua, *Theor. Chem. Acc.* **2012**, *131*, 1129.

[6] A. Padua, Force field for ionic liquids https://github.com/agiliopadua/ilff. https://doi.org/10.5281/zenodo.593705.

[7] J. L. F. Abascal, C. Vega, *J. Chem. Phys*. **2005**, *123*, 234505.

[8] R. W. Hockney, J. W. Eastwood, Computer Simulation Using Particles. Bristol: Hilger, **1988**.

[9] W. G. Hoover, *Phys. Rev.* **1985**, *31*, 1695.
